# Resolving the contrasting leaf hydraulic adaptation of C_3_ and C_4_ grasses

**DOI:** 10.1111/nph.20341

**Published:** 2025-01-05

**Authors:** Alec S. Baird, Samuel H. Taylor, Jessica Pasquet‐Kok, Christine Vuong, Yu Zhang, Teera Watcharamongkol, Hervé Cochard, Christine Scoffoni, Erika J. Edwards, Colin P. Osborne, Lawren Sack

**Affiliations:** ^1^ Department of Ecology and Evolutionary Biology University of California Los Angeles 621 Charles E. Young Dr. South Los Angeles CA 90095 USA; ^2^ Institute of Plant Sciences University of Bern Altenbergrain 21 3013 Bern Switzerland; ^3^ Oeschger Centre for Climate Change Research University of Bern Bern 3012 Switzerland; ^4^ Lancaster Environment Centre University of Lancaster Lancaster LA1 4YW UK; ^5^ Plants, Photosynthesis and Soil, School of Biosciences University of Sheffield Sheffield S10 2TN UK; ^6^ Faculty of Science and Technology Kanchanaburi Rajabhat University Kanchanaburi 71190 Thailand; ^7^ Université Clermont Auvergne, INRAE, PIAF 63000 Clermont‐Ferrand France; ^8^ Department of Biological Sciences California State University Los Angeles 5151 State University Dr. Los Angeles CA 90032 USA; ^9^ Department of Ecology and Evolutionary Biology Yale University New Haven CT 06520 USA

**Keywords:** aridity, climate, drought tolerance, photosynthesis, Poaceae, water transport

## Abstract

- Grasses are exceptionally productive, yet their hydraulic adaptation is paradoxical. Among C_3_ grasses, a high photosynthetic rate (*A*_area_) may depend on higher vein density (*D*_v_) and hydraulic conductance (*K*_leaf_). However, the higher *D*_v_ of C_4_ grasses suggests a hydraulic surplus, given their reduced need for high *K*_leaf_ resulting from lower stomatal conductance (*g*_s_).
- Combining hydraulic and photosynthetic physiological data for diverse common garden C_3_ and C_4_ species with data for 332 species from the published literature, and mechanistic modeling, we validated a framework for linkages of photosynthesis with hydraulic transport, anatomy, and adaptation to aridity.
- C_3_ and C_4_ grasses had similar *K*_leaf_ in our common garden, but C_4_ grasses had higher *K*_leaf_ than C_3_ species in our meta‐analysis. Variation in *K*_leaf_ depended on outside‐xylem pathways. C_4_ grasses have high *K*_leaf_ : *g*_s_, which modeling shows is essential to achieve their photosynthetic advantage.
- Across C_3_ grasses, higher *A*_area_ was associated with higher *K*_leaf_, and adaptation to aridity, whereas for C_4_ species, adaptation to aridity was associated with higher *K*_leaf_ : *g*_s_. These associations are consistent with adaptation for stress avoidance.
- Hydraulic traits are a critical element of evolutionary and ecological success in C_3_ and C_4_ grasses and are crucial avenues for crop design and ecological forecasting.

## Introduction

The grass family (Poaceae) dominates > 40% of the Earth's terrestrial surface with 12 000 species from 800 genera, including the bulk of all crops (Beer *et al*., 2010; McSteen & Kellogg, 2022). The photosynthetic diversity of grasses is a major factor in their dominance and in their resilience to climate change (Higgins & Scheiter, 2012). More than 40% of extant grass species have C_4_ photosynthesis, which evolved > 20 times in grasses (of the > 60 times across angiosperms) and is a model for the repeated emergence of a key innovation (Gowik & Westhoff, 2011; Sage *et al*., 2011; Grass Phylogeny Working Group II, 2012; Marazzi *et al*., 2012), and the source of high yield in many crops and for novel varieties in development (Gowik & Westhoff, 2011; Langdale, 2011). C_4_ photosynthesis maximizes carbon fixation, particularly under hotter, drier conditions or low CO_2_, by concentrating CO_2_ at Rubisco in the sheath around the leaf veins, minimizing photorespiratory losses, and enabling reduced stomatal conductance per leaf area (*g*_s_) and higher light‐saturated photosynthetic rate per leaf area (*A*_area_) relative to *g*_s_, resulting in higher intrinsic water use efficiency (WUE_i_, that is *A*_area_ : *g*_s_) (Supporting Information Table S1) (Sage, 2004). Yet, there has been only a fragmentary understanding of the potential contrasts in leaf hydraulic design underlying the photosynthetic and climate adaptation of C_3_ and C_4_ grasses, though previous work on grass leaf hydraulic design has indicated its importance in C_3_ and C_4_ grass performance (Ocheltree *et al*., 2014; Baird *et al*., 2021; Zhou *et al*., 2021).

**Fig. 1:**
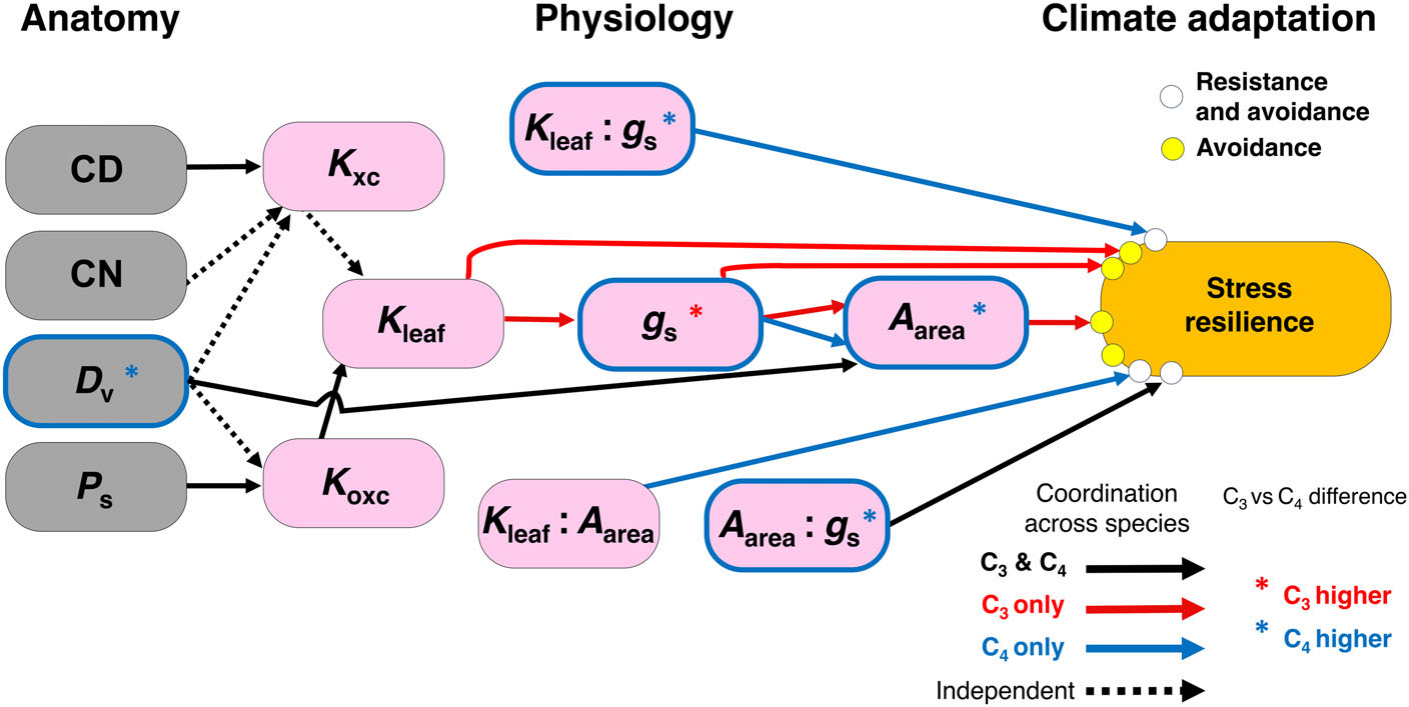
Conceptual framework linking leaf anatomical, hydraulic and gas exchange traits, and their coordinated influence on adaptation to climatic aridity, supported in a common garden experiment including 27 C_3_ and C_4_ species. Gray and pink nodes represent anatomical and physiological traits, respectively, that contribute to drought resilience (orange node). Arrows represent relationships expected from hydraulic theory (Table 1) and supported by our experimental data. According to Hypothesis 1: a higher ratio of leaf hydraulic conductance to stomatal conductance, *K*_leaf_ : *g*_s_ (whether driven by a high *K*_leaf_ or low *g*_s_), in C_4_ grasses would enable the maintenance of higher leaf water potential and *g*_s_ at lower soil water potential and/or higher vapor pressure deficit, enabling the realization of the biochemical advantage of C_4_, that is, high light‐saturated photosynthetic rate per leaf area (*A*_area_). According to Hypotheses 2–3: among C_3_ grasses, a high *K*_leaf_ enables a higher *g*_s_, by maintaining high leaf water potential at a given transpiration rate and vapor pressure deficit, in turn enabling higher *A*_area_. Among C_4_ grasses, the disproportionately high *K*_leaf_ : *g*_s_ leads to decoupling of *K*_leaf_ from *g*_s_. According to Hypotheses 4–8: across all species, higher mean leaf vein conduit diameter (CD), conduit number (CN), and/or vein density (*D*_v_) would contribute to higher vein xylem conduit hydraulic conductance (*K*_xc_), and a higher *D*_v_ and/or vein sheath perimeter (*P*_s_) to higher outside‐xylem conduit hydraulic conductance (*K*_oxc_); a higher *K*_xc_ or *K*_oxc_ would drive higher *K*_leaf_. Additionally, a higher *D*_v_ may provide greater sugar transport capacity and thereby be linked with higher *A*_area_. According to Hypotheses 9–10: multiple traits would contribute to drought resilience, that is, via drought resistance (the ability to maintain effective function during drought), including higher *K*_leaf_ : *g*_s_, *K*_leaf_ : *A*_area_, and *A*_area_ : *g*_s_ for C_4_ grasses; and drought avoidance (the ability to mitigate the impact of drought with high performance when moisture is available), including higher *K*_leaf_, *g*_s_, *A*_area_ among C_3_ and higher *K*_leaf_ : *g*_s_ and *K*_leaf_ : *A*_area_ for C_4_, and higher *A*_area_ : *g*_s_ among C_3_ and C_4_, and thus adaptation to arid climates. Significant relationships in common garden‐grown plants are depicted by solid arrows, black if significant across C_3_ and C_4_ species combined, red if significant for C_3_ only, and blue for C_4_ only; dotted lines indicate that traits that in theory (all else being equal) would contribute mechanistically to other traits, yet in this study were statistically decoupled across the studied species. Traits depicted with blue borders differed in our study on average between C_3_ and C_4_ species, and would contribute to the C_4_ advantage; asterisks in red or blue indicate a higher value for C_3_ or C_4_ species, respectively. This framework is strictly conceptual and based on the hypothesized mechanisms in Table 1.

Generally, across plants, the leaves are bottlenecks in water transport and impose a major limitation on photosynthetic productivity (Meinzer *et al*., 1992; Martre *et al*., 2000; Sack & Holbrook, 2006). We extended the theory for the dependence of leaf gas exchange on leaf hydraulic anatomy and physiology established across diverse C_3_ angiosperms (Sack & Holbrook, 2006; Brodribb *et al*., 2007) by hypothesizing a novel general framework for the contrasting adaptation of C_3_ and C_4_ grasses (Fig. 1; Table 1). The premise of this theory is that water supply through the integrated leaf system needs to match evaporative demand for leaf water potential (Ψ_leaf_) to be maintained high enough for stomata to open for photosynthetic CO_2_ assimilation (Sack & Holbrook, 2006). During transpiration, liquid water moves through the network of leaf veins, which have high density (i.e. length per leaf area, *D*_v_), and then across the bundle sheath and through the mesophyll to the sites of evaporation before diffusion from the stomata (Sack & Scoffoni, 2013), and the capacity of water transport through this system is quantified as the leaf hydraulic conductance (*K*_leaf_), the ratio of transpiration rate to water potential driving force. Accordingly, across plant life forms and closely related C_3_ angiosperms, hydraulics and gas exchange traits such as *D*_v_, *K*_leaf_, *g*_s_, and *A*_area_ are positively coordinated (Brodribb *et al*., 2007; Scoffoni *et al*., 2016).

**Table 1:** Framework of hypotheses for the contrasting hydraulic adaptation of C_3_ and C_4_ grasses, with reasoning and synthesis from previous studies.

Hypothesis	Previous work and rationale
*Contrasting basis for photosynthetic diversity in C*_*3*_ *and C*_*4*_ *grasses, and C*_*4*_ *hydraulic hyper‐efficiency*	
1. High photosynthetic capacity of C_3_ grasses depends on high leaf hydraulic conductance (*K*_leaf_), and in C_4_ grasses on high *K*_leaf_ relative to stomatal conductance (*g*_s_), i.e. *K*_leaf_ : *g*_s_, enabling its high photosynthetic rate (*A*_area_) and *A*_area_ : *g*_s_ (i.e. high intrinsic water use efficiency, WUE_i_). A high *K*_leaf_ in C_3_ grasses would enable high *g*_s_ and thereby higher *A*_area_, whereas a higher *K*_leaf_ : *g*_s_ in C_4_ grasses (i.e. hydraulic hyper‐efficiency) would enable higher operating leaf water potential (Ψ_leaf_), vital for realizing their higher gas exchange rates, especially necessary given the strong sensitivity of C_4_ biochemistry to declining Ψ_leaf_ (Ghannoum *et al*., 2003; Osborne & Sack, 2012; Taylor *et al*., 2014; Bellasio *et al*., 2023).	In the six previous studies of hydraulic capacity in C_3_ vs C_4_ species of grasses or eudicots, contrasting results were reported. In three studies, *K*_leaf_ was similar for C_3_ and C_4_ grass species, that is, for studies of temperate grasses (Ocheltree *et al*., 2014), of subtropical perennial grasses (Liu *et al*., 2019) and annual grasses used as crops or their close relatives (Taylor *et al*., 2018). In two studies, *K*_leaf_ was higher for C_4_ than C_3_ grass species, that is, for studies of subtropical annual grasses (Liu *et al*., 2019) and temperate and tropical grasses, and annual and perennial grasses (Zhou *et al*., 2021). In one study, *K*_leaf_ was lower in C_4_ *Panicum antidotale* relative to its C_3_ sister taxon *P. bisulcatum* (Sonawane *et al*., 2021). In studies of C_3_ and C_4_ eudicots, temperate herbaceous C_4_ species had lower stem hydraulic conductance (Kocacinar & Sage, 2003) as did temperate woody C_4_ species (Kocacinar & Sage, 2004). In one study, temperate C_3_ grass species had higher *K*_leaf_ than tropical C_4_ species (Jacob *et al*., 2022). A high *K*_leaf_ : *g*_s_ enables the maintenance of *g*_s_ under atmospheric drought (i.e. high vapor pressure deficits) for temperate and tropical tree species (Brodribb & Jordan, 2008; Scoffoni *et al*., 2016). High *K*_leaf_ : *g*_s_ was hypothesized to enable the evolution of C_4_ photosynthesis under drying conditions in a low CO_2_ past (Osborne & Sack, 2012).
*Contrasting coordination of hydraulic*, *stomatal and photosynthetic function in C*_*3*_ *and C*_*4*_ *grasses*	
2. Across C_3_ grasses *K*_leaf_, *g*_s_ and *A*_area_ are positively coordinated. *A*_area_ would show a saturation response to higher *g*_s_ across C_3_ grasses.	Previous studies of diverse species (Brodribb *et al*., 2007), C_3_ eudicotyledons (Scoffoni *et al*., 2016), and grasses (Zhou *et al*., 2021) showed a positive coordination of hydraulics and gas exchange, that is, of *K*_leaf_, stomatal conductance (*g*_s_), and light‐saturated photosynthetic rate per unit leaf area (*A*_area_). The high mesophyll resistance to CO_2_ diffusion in C_3_ leaves would lead to saturating effects of *A*_area_ at high *g*_s_ (von Caemmerer & Evans, 2010).
3. Across C_4_ grasses, *g*_s_ and *A*_area_ are decoupled from *K*_leaf_. *A*_area_ would increase linearly with *g*_s_ across C_4_ grasses.	In C_4_ grasses, selection for high WUE_i_, and thus, low *g*_s_ would result in a decoupling of *K*_leaf_ and *g*_s_ (Zhou *et al*., 2021). Decoupling of *K*_leaf_ and *g*_s_ was previously shown separately across 18 and nine C_4_ grasses (Ocheltree *et al*., 2016; Pathare *et al*., 2020), and for *K*_leaf_ and *A*_area_ across 29 C_4_ grasses (Zhou *et al*., 2021). A linear scaling of *A*_area_ with *g*_s_ is expected for C_4_ species, which indicates a low role for mesophyll resistance in constraining photosynthetic rate (Bjorkman, 1971).
*Contrasting anatomical drivers of grass leaf hydraulic function*	
4. Across C_3_ and C_4_ grasses, variation in *K*_leaf_ depends on outside‐xylem conduit hydraulic conductance (*K*_oxc_) rather than xylem conduit hydraulic conductance (*K*_xc_).	The parallel vein system of grasses, containing large xylem conduits, would provide high axial xylem transport efficiency (Givnish, 1979) such that *K*_oxc_ would more strongly constrain *K*_leaf_ across species. A large bottleneck to water transport outside the xylem was reported for nine rice genotypes (Xiong *et al*., 2017).
5. Variation in *K*_xc_ is driven by variation in xylem conduit diameter (CD) across C_3_ and C_4_ grasses.	Hydraulic conductance is highly sensitive to conduit diameter, with a 4^th^ power dependency according to the Hagen–Poiseuille equation (Nobel, 2020).
6. Across C_3_ and C_4_ grasses, variation in *K*_oxc_ is driven by variation in vein sheath properties.	A higher vein sheath perimeter represents a greater surface for exchange with surrounding mesophyll symplast and apoplast, and thus more membrane aquaporins, plasmodesmata, and cell wall transport pathways beyond suberin and lignin barriers, and would increase hydraulic conductance (Mertz & Brutnell, 2014; Sade *et al*., 2015).
7. Higher major vein density (*D*_v‐major_) and surface area per area (*VSA*_major_) drive higher *K*_leaf_ and/or *A*_area_ in C_3_ grasses.	*D*_v‐major_ and *VSA*_major_ may influence *K*_oxc_, *K*_leaf_ and/or *A*_area_. The major veins transport the bulk of leaf water throughout the leaf, given their large xylem conduits, and their larger surface for radial delivery of water to the mesophyll in transpiring leaves. These major vein traits may also correspond to greater sugar transport capacity in the phloem, and this too would be linked with higher *A*_area_ (Adams *et al*., 2013).
8. In C_4_ species, a higher *D*_v_ would not drive a higher *K*_leaf_ but would drive a higher *A*_area_ given its representing greater allocation to vein sheath carbon assimilation tissue.	Across grasses, which have parallel major veins containing large xylem conduits, the minor vein traits (and *D*_v_, which is related most strongly to minor vein density) would contribute minimally to the overall determination of *K*_xc_ or to *K*_leaf_, and potentially a higher *D*_v_ would not entail substantially greater *K*_xc_ relative to construction costs, if it were linked with reduce conduit numbers and/or sizes. The higher minor *D*_v_ of C_4_ grasses (Ueno *et al*., 2006; Baird *et al*., 2021) reflects greater allocation to vein sheaths, that is, to Kranz anatomy, and thus to carbon assimilation in sheath cells.
*Contrasting adaptation of leaf hydraulics and gas exchange traits to climate in C*_*3*_ *and C*_*4*_ *grasses*	
9. In C_3_ grasses, adaptation to aridity depends on higher *K*_leaf_, *g*_s_ and *A*_area_.	C_3_ grasses would adapt to aridity with higher hydraulic and photosynthetic rates, providing drought avoidance, that is, an ability to mitigate stressful periods by growing rapidly when water is abundant (Grubb, 1998; Volaire, 2018; Fletcher *et al*., 2022).
10. In C_4_ grasses, adaptation to aridity depends on higher *K*_leaf_ : *g*_s_.	C_4_ grasses would adapt to aridity with a higher hydraulic supply relative to demand, providing both drought resistance, that is, an ability to maintain gas exchange when soil moisture is low, and drought avoidance, that is, growing rapidly when water is abundant (Grubb, 1998; Volaire, 2018; Fletcher *et al*., 2022).

See Supporting Information Table S1 for trait definitions and units.

Hydraulic adaptations depend strongly on anatomy. A higher *K*_leaf_ can arise from a greater conductance of the xylem conduits, and/or of the outside‐xylem conduit pathways (*K*_xc_ and *K*_oxc_, respectively):

(Eqn 1) $$K_{\text{leaf}}={\left(K_{\mathrm{xc}}^{-1}+K_{\mathrm{oxc}}^{-1}\right)}^{-1}$$

A higher *K*_xc_ can be achieved through vein xylem traits, including higher conduit diameter (CD), conduit number (CN), and/or a higher *D*_v_, which represents more parallel flow pathways. A higher *K*_oxc_ can also be achieved through higher *D*_v_, shortening outside‐xylem flow pathways, and also through traits that would increase vein sheath conductance (Sack & Scoffoni, 2013) (Fig. 1).

We extended hypotheses for the centrality of hydraulic adaptation in the evolution of C_4_ photosynthesis in grasses. The C_4_ carbon concentrating mechanism enables a higher *A*_area_ despite lower *g*_s_, and higher operating Ψ_leaf_ (Osborne & Freckleton, 2009; Taylor *et al*., 2010, 2011, 2014; Zhou *et al*., 2021). The evolution of high photosynthetic rates in C_4_ grasses depended on high *D*_v_ and enlarged mestome and/or bundle sheath cells, reducing the distance between mesophyll and sheath cells, thereby enabling the development of ‘Kranz’ anatomy for rapid movement of metabolites between mesophyll and sheath cells (Ogle, 2003; Sage, 2004; Ueno *et al*., 2006; Christin *et al*., 2013; Baird *et al*., 2021). Yet, unlike C_3_ grasses, C_4_ species, once having evolved a lower *g*_s_, may not require a higher *K*_leaf_ to achieve higher rates of gas exchange (Fig. 1). Indeed, for C_4_ eudicots, stem hydraulic conductance was reduced relative to C_3_ relatives (Kocacinar & Sage, 2003, 2004). Thus, the lack of a requirement for a high *K*_leaf_ in C_4_ grasses poses an unresolved anatomical paradox. In C_4_ grasses, the typically higher *D*_v_ associated with Kranz anatomy presents an apparent surplus of hydraulic capacity, given their reduced need for *K*_leaf_ due to lower *g*_s_ (Ueno *et al*., 2006; Baird *et al*., 2021). However, if higher *D*_v_ were coupled with fewer xylem conduits within these veins, this may negate impacts on *K*_leaf_, and also indicate little carbon cost constraints to evolving higher *D*_v_. C_4_ grasses might thus be an exception to the specific trends observed across diverse plant lineages for the association between hydraulic and photosynthetic traits and their adaptation to climate. A previously proposed, but untested, hypothesis is that C_4_ grasses would tend to have a higher *K*_leaf_ relative to *g*_s_ than C_3_ species, enabling the C_4_ species to maintain higher Ψ_leaf_ under mild to moderate soil or atmospheric drought that would otherwise drive declining *A*_area_ (Taylor *et al*., 2011; Osborne & Sack, 2012) (Table 1). A high *K*_leaf_ : *g*_s_ was hypothesized to enable the evolution of C_4_ photosynthesis under drying conditions, especially in a low CO_2_ past (Osborne & Sack, 2012), yet data have not been available to test this hypothesis. The importance of a high *K*_leaf_ : *g*_s_ may be especially necessary given that C_4_ biochemistry is highly sensitive to declining Ψ_leaf_ (Ghannoum *et al*., 2003; Bellasio *et al*., 2023). In contrast, we hypothesized that *D*_v_, *K*_leaf_, *g*_s_, and *A*_area_ would be positively coordinated across C_3_ grass species, as shown across diverse major plant lineages and across closely related angiosperms (Brodribb *et al*., 2007; Scoffoni *et al*., 2016).

We hypothesized that contrasting hydraulic traits of C_3_ and C_4_ grasses would result in differential climatic stress adaptation. Resilience to stress can depend on traits contributing to stress tolerance (i.e., maintaining growth throughout a period that includes a stress). The ability to recover after stress and, in turn, stress tolerance can be achieved through stress resistance (i.e. maintenance of function during stress), and/or avoidance (i.e. relative dormancy during stress, and maximizing growth during warm and wet periods) (Hodgson *et al*., 2015; Volaire, 2018; Fletcher *et al*., 2022). For C_3_ grasses, high *A*_area_ is associated with higher *g*_s_ and transpiration rate per leaf area, and thus with high water demand, therefore requiring a higher *K*_leaf_ and also greater extraction of soil water. These traits would contribute to stress avoidance, that is, the maximization of assimilation under high water availability and the ability to cope with stressful periods through dormancy or an annual life cycle. By contrast, certain traits would contribute to both stress resistance and avoidance, including higher *A*_area_ : *g*_s_ (WUE_i_) and higher *K*_leaf_ : *g*_s_, as these would enable high photosynthetic returns during dry and also moist periods, mediated by higher operating leaf water potential (Hodgson *et al*., 2015; Volaire, 2018; Fletcher *et al*., 2022).

We hypothesized a contrasting coordination of hydraulic, stomatal, and photosynthetic traits in C_3_ and C_4_ species that contributes to their ecological differentiation along a gradient of aridity. We tested a framework of hypotheses (Table 1; Fig. 1) using experimental data for > 30 traits from a common experimental garden of 11 C_3_ and 16 C_4_ grass species, including species native to diverse habitats and major crops. With respect to phylogeny, our sample included representatives of 11 independent C_4_ origins and 5 sister C_3_ clades sampled within the PACMAD, as well as outgroup C_3_ comparators from Oryzoideae and Pooideae (Fig. S1; Tables S1, S2). Additionally, we meta‐analyzed a compiled database with data from 37 previously published studies for a total of 332 species from field studies and common garden experiments (Table S3). We elucidated the variation in *K*_leaf_ and its components for C_3_ and C_4_ grasses, their anatomical determinants, and the coordination of hydraulic and gas exchange traits with adaptation to aridity (Fig. 1; Table 1).

## Materials and Methods

### Plant material for experimental common garden of C_3_ and C_4_ grasses

We grew 27 species selected to capture large functional and phylogenetic diversity, including 11 and 16 C_3_ and C_4_ species, respectively, representing 11 independent C_4_ origins, and five C_3_ sister clades within the PACMAD (Fig. S1; Table S2), and utilized phylogenetically matched contrasts of closely related species (Funk *et al*., 2015). Growing conditions are described in previous studies based on this experiment (Baird *et al*., 2021, 2024) and also summarized in Methods S1. Plants were grown in a common garden design at the UCLA Plant Growth Center to reduce environmentally driven plasticity that occurs across species' distributions in the wild and thereby to better resolve genetic adaptation (Cordell *et al*., 1998; Givnish & Montgomery, 2014; Huxman *et al*., 2022).

We included in our analyses previously published data (Baird *et al*., 2021, 2024) for a number of vein traits (i.e. vein diameter (VD), vein density (*D*_v_), vein surface area per leaf area (VSA), vein projected area per leaf area (VPA), vein volume per leaf area (VVA), maximum conduit diameter (CD), leaf thickness (LT), and light‐saturated photosynthetic rate per leaf area (*A*_area_), as well as species‐level climate data (mean annual temperature (MAT), mean annual precipitation (MAP), and mean annual aridity index (AI), i.e. MAP/potential evapotranspiration (PET)). Other hydraulic, morphological, and anatomical traits are novel to this study (described below) and were measured over the same several‐month period or from tissues sampled at that time from the same plants evaluated in the previous studies (Baird *et al*., 2021, 2024).

### Sample anatomical preparation

Following the establishment of at least 3–4 mature leaves, one leaf from each of three individuals per species was fixed and stored in FAA solution (37% formaldehyde–glacial acidic acid‐95% ethanol in deionized water). Leaf samples were used for creation of transverse cross sections (Methods S2).

### Quantification of leaf hydraulic traits

We measured the leaf hydraulic conductance (*K*_leaf_) using the steady‐state evaporative flux method (EFM) (Sack & Scoffoni, 2012), for 2–3 leaves per plant from six plants, resulting in 6–18 leaves per species (Methods S3). We determined *K*_leaf_ by averaging all *K*_leaf_ measurements for each species.

We estimated hydraulic vulnerability as the Ψ_leaf_ at 50% loss of *K*_leaf_ (*P*_50_). For the 23 species, a linear regression fitted the data (*R* ^2^ = 0.40–0.88; *P* < 0.001–0.019, ordinary least squares using SMATR; Warton *et al*., 2012), allowing identification of Ψ_leaf_ at which *K*_leaf_ declined to half of the *y*‐intercept value. For numerous species including grasses, a straight line approximates the decline at high Ψ_leaf_ (Pasquet‐Kok *et al*., 2010; Holloway‐Phillips & Brodribb, 2011; Scoffoni *et al*., 2012).

We determined hydraulic to stomatal conductance ratio as the ratio of mean leaf hydraulic conductance relative to stomatal conductance (*K*_leaf_ : *g*_s_).

Using *K*_xc_ determined by anatomical measurements (see the Quantification of vein xylem, and sheath anatomical traits subsection below), we determined *K*_oxc_ by re‐arranging Eqn 1:

$$K_{\mathrm{oxc}}={\left(K_{\text{leaf}}^{-1}-K_{\mathrm{xc}}^{-1}\right)}^{-1}$$

### Quantification of leaf gas exchange

We measured steady‐state light‐saturated rates of gas exchange (< 2% change over 6 min) from 17 February to 28 June 2010, between 09:00 and 15:00 h each day, for a mature leaf on each plant for six plants per species using a LI‐6400 XT portable photosynthesis system (Li‐Cor, Lincoln, Nebraska, USA) (Methods S4). These measurements represented maximum rates of gas exchange, which did not differ significantly across the time of each day measured. Vapor pressure deficits (VPD) in the chamber were 0.80–1.6 kPa and the chamber was maintained at 25°C.

### Quantification of vein xylem, and sheath anatomical traits

We measured and analyzed cross sections of one leaf for each of three individuals per species (Methods S5).

Vein conduit dimensions and numbers were measured for one leaf per individual for three individuals per species, in one vein of each vein order in each leaf from transverse sections imaged under a ×40 objective using a light microscope (Leica Lietz DMRB; Leica Microsystems) and camera with imaging software (SPOT Imaging Solution; Diagnostic Instruments, Sterling Heights, Michigan USA). Xylem conduits were identified by toluidine blue staining of the lignified cell walls. The theoretical conductivity (*k*_t_; mmol m s^−1^ MPa^−1^) was determined from Poiseuille's equation modified for ellipses (Lewis & Boose, 1995; Cochard *et al*., 2004; Scoffoni *et al*., 2016),

(Eqn 2) $$k_{t}=\frac{\pi }{64\mu }\frac{a^{3}b^{3}}{a^{2}+b^{2}}$$

where *μ* is the viscosity of water at 25°C, and *a* and *b* are the major and minor axes of the ellipse, respectively. We measured *a* and *b* for all xylem conduits and averaged this estimate of conduit diameter for all conduits within a given vein order for each type. In grass leaves, protoxylem conduits form early within major vein orders and are destroyed during leaf expansion, which results in an empty space termed the protoxylem lacuna (Evert, 2006). We measured the dimensions of the protoxylem lacunae, as this space also transports water (Buchholz, 1921; Canny, 2001), and the wide and narrow xylem conduits (xylem type I and II, respectively) within the major veins, and the narrow xylem conduits within the minor veins (xylem II). The *k*_t_ of each longitudinal vein order was determined as the sum of the *k*_t_ of all conduits of all types:

(Eqn 3) $$1{}^{\circ}\;k_{t\;}=1{}^{\circ}\;k_{t}\;\text{Xylem}\;I+1{}^{\circ}\;k_{t}\;\text{Xylem}\;\mathrm{II}+1{}^{\circ}\;k_{t}\;\text{Protoxylem Lacuna}$$

(Eqn 4) $$2\;{}^{\circ}k_{t}=2{}^{\circ}\;k_{t}\;\text{Xylem}\;I+2{}^{\circ}\;k_{t}\;\text{Xylem}\;\mathrm{II}+2{}^{\circ}\;k_{t}\;\text{Protoxylem Lacuna}$$

(Eqn 5) $$3{}^{\circ}\;k_{t}=3{}^{\circ}\;k_{t}\;\text{Xylem}\;\mathrm{II}$$

(Eqn 6) $$4{}^{\circ}\;k_{t}=4{}^{\circ}\;k_{t}\;\text{Xylem}\;\mathrm{II}$$

where $k_{t}\;\text{Xylem}\;I$ is the summed *k*_t_ of all type I xylem conduits, $k_{t}\;\text{Xylem}\;\mathrm{II}$ is the summed *k*_t_ of all type II xylem conduits, and *k*_t_ Protoxylem Lacuna is the *k*_t_ of the single protoxylem lacuna. This approach to the estimation of the theoretical xylem conductance (*k*_t_) is highly standard in the field and has been used for wood (Weitz *et al*., 2006; Alber *et al*., 2019), veins (Sack & Frole, 2006; Pasquet‐Kok *et al*., 2010; Sommerville *et al*., 2012; Gleason *et al*., 2016; North *et al*., 2016; Scoffoni *et al*., 2016), and grasses (Martre *et al*., 2001; Martre & Durand, 2001).

**Fig. 2:**
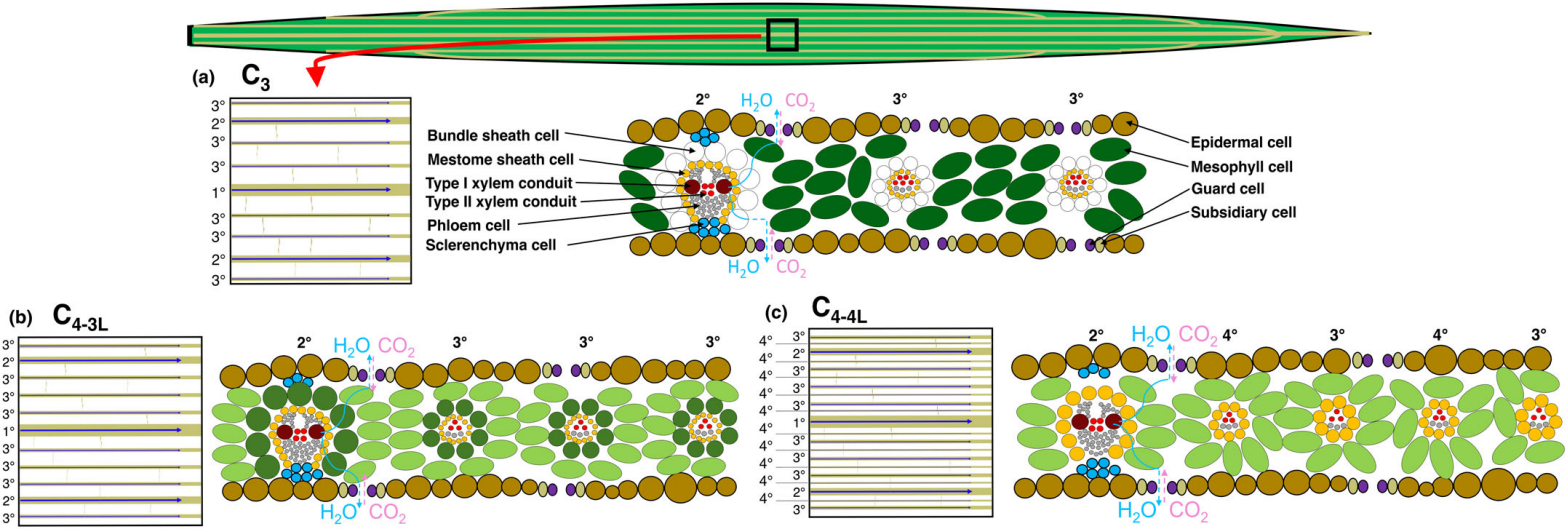
Leaf hydraulic anatomy of grasses. Grasses have linearized leaves in which water flows through up to four orders of parallel longitudinal veins, including the 1° midvein and large 2° major veins, intermediate 3° minor veins and, in C_4_ NADP‐ME species of the subfamily Panicoideae, small 4° veins, all connected by 5° transverse veins. Water then flows outside the xylem, radially across vein sheaths, which often have hydrophobic cell walls due to suberization and/or lignification, including the mestome sheath (MS) interior to the bundle sheath (BS), and through the mesophyll, before evaporating and diffusing out of the leaf. (a) C_3_ and (b) most C_4_ species (i.e. C_4‐3L_) possess three longitudinal vein orders, whereas (c) most C_4_ species of the subfamily Panicoideae evolved an additional fourth vein order, in which the MS is the only sheath (i.e. C_4‐4L_). Carbon reduction reactions (depicted with dark green) occur in (a) mesophyll of C_3_ species, (b) in the BS of C_4‐3L_ species and (c) in the MS in C_4‐4L_ species, which is depicted in orange to differentiate the developmental origin of the MS from procambium tissue, in contrast with the BS, which derives from nonprocambium tissue (c); C_4_ grasses have higher total and minor *D*_v_, higher bundle and mestome sheath diameters (Christin *et al*., 2013), and lower stomatal densities (Taylor *et al*., 2012). The red arrow indicates a subpanel of grass leaf venation. Black arrows indicate cell types of grass leaves. Blue and pink arrows indicate water and carbon dioxide, respectively.

We calculated whole‐leaf *k*_t_ (mmol m s^−1^ MPa^−1^) by summing the *k*_t_ values for each longitudinal parallel vein order (Fig. 2):

(Eqn 7) $$k_{t}=1{}^{\circ}k_{t}+2{}^{\circ}k_{t}+3{}^{\circ}k_{t}+4{}^{\circ}k_{t}$$

For estimating *K*_xc_ from vein anatomy, we applied a widely used approach (Sack & Frole, 2006; Pasquet‐Kok *et al*., 2010; Sommerville *et al*., 2012; Gleason *et al*., 2016; North *et al*., 2016; Scoffoni *et al*., 2016) (Methods S6) that has been shown to match measured *K*_xc_ values for grasses (Xiong *et al*., 2017). We calculated a leaf length and area‐normalized conductance of the xylem conduit lumen component of the vein system (*K*_xc_, mmol m^−2^ s^−1^ MPa^−1^) by multiplying the *k*_t_ of each vein order by its vein density (*D*_v_, that is vein length per leaf area), which is equivalent to vein number per width for grasses, excluding transverse veins (Baird *et al*., 2021), and then dividing by half the leaf length (LL) squared. Normalizing by LA and LL is necessary to scale the *K*_t_ from a conductivity to an area‐specific conductance (Pasquet‐Kok *et al*., 2010); using half the leaf length yields a *K*_xc_ representing the average vein hydraulic pathway, assuming that longitudinal veins deliver water similarly along their length, on average. *K*_xc_ normalized by length and area in this way is in the same units as *K*_leaf_:

(Eqn 8) $$K_{\mathrm{xc}}=\begin{array}{l} ((\left(1{}^{\circ}\;k_{t}\times 1{}^{\circ}\;D_{V}\right)+\left(2{}^{\circ}\;k_{t}\times 2{}^{\circ}\;D_{V}\right)+\left(3{}^{\circ}\;k_{t}\times 3{}^{\circ}\;D_{V}\right)\\ +\left(4{}^{\circ}\;k_{t}\times 4{}^{\circ}\;D_{V}\right))÷\left(0.5\times \left({\mathrm{LL}}^{2}\right)\right))÷0.71 \end{array}$$

where 0.71 is a leaf shape area correction factor for linear leaves (Shi *et al*., 2019; Schrader *et al*., 2021).

We estimated the xylem construction cost of the major, minor, and whole vein architecture, using an index of cell wall volume per leaf area (CC; McKown *et al*., 2010).

CC for a given vein order was estimated as:

(Eqn 9) $$\mathrm{CC}=\pi \times \mathrm{CD}\times \mathrm{CN}\times D_{v}$$

where CD is the conduit diameter of the vein order, CN is the conduit number of the vein order, and *D*_v_ is the vein density of the vein order. For this estimation of CC, we considered xylem conduits wall thickness to be a constant (McKown *et al*., 2010). Recent work reported that, on average across woody dicot species, wider conduits have thicker (but proportionally thinner) walls (Matos *et al*., 2024); in that case, the CD term in Eqn 9 would be raised to an exponent < 1, and we considered that derivation in our interpretation.

We estimated anatomical traits as correlates of leaf outside‐xylem conduit hydraulic conductance (*K*_oxc_) (Methods S7). As an estimate of the surface available for flow out of the vasculature to the mesophyll, we quantified the outer perimeter of the bundle and mestome sheath (*P*_bs_ and *P*_ms_) layers for all vein orders (Fig. 2). For each vein order, we measured the diameter of the major and minor axes of one small, medium, and large bundle and/or mestome sheath cell and averaged the major and minor axis diameters per cell, and then averaged across the cell size classes to obtain an average cell diameter. To estimate the outer perimeter, we divided this average cell diameter (*D*) by two and multiplied by π (i.e., representing half the perimeter of a circle) and by the number of bundle or mestome sheath cells (*N*) surrounding the vein of a given order and then averaged this value across all vein orders:

(Eqn 10) $$P_{\mathrm{bs}}=\left(\left(\left(1{}^{\circ}D_{\mathrm{bs}}÷2\right)\times \pi \times 1{}^{\circ}N_{\mathrm{bs}}\right)+\left(\left(2{}^{\circ}D_{\mathrm{bs}}÷2\right)\times \pi \times 2{}^{\circ}N_{\mathrm{bs}}\right)+\left(\left(3{}^{\circ}D_{\mathrm{bs}}÷2\right)\times \pi \times 3^{{}^{\circ}}\;N_{\mathrm{bs}}\right)\right))÷3$$

(Eqn 11) $$P_{\mathrm{ms}}=\left(\left(\left(1{}^{\circ}D_{\mathrm{ms}}÷2\right)\times \pi \times 1{}^{\circ}N_{\mathrm{ms}}\right)+\left(\left(2{}^{\circ}D_{\mathrm{ms}}÷2\right)\times \pi \times 2{}^{\circ}N_{\mathrm{ms}}\right)+\left(\left(3{}^{\circ}D_{\mathrm{ms}}÷2\right)\times \pi \times 3{}^{\circ}N_{\mathrm{ms}}\right)+\left(\left(4{}^{\circ}D_{\mathrm{ms}}÷2\right)\times \pi \times 4{}^{\circ}N_{\mathrm{ms}}\right)\right))÷4$$

We also estimated the bundle and mestome sheath surface area per leaf area (BSSA and MSSA), projected area per leaf area (BSPA and MSPA) and volume per leaf area (BSV and MSV) for each vein order, and we present total BSSA and MSSA, BSPA and MSPA, and BSV and MSV (i.e., sum of all vein order bundle and mestome sheath surface areas, projected areas, or volumes), major BSSA and MSSA, BSPA and MSPA, and BSV and MSV (i.e., sum of major vein bundle and mestome sheath surface areas, projected areas, or volumes), and minor BSSA and MSSA, BSPA and MSPA, and BSV and MSV (i.e., sum of minor vein bundle and mestome sheath surface areas, projected areas, or volumes). We estimated the BSSA and MSSA of each vein order by first multiplying the average bundle or mestome sheath cell diameter (*D*) (as mentioned in the previous section) by the *D*_v_ of the vein order and by π and by the number of cells present (*N*), the BSPA and MSPA by multiplying the average bundle or mestome sheath cell diameter (*D*) (as mentioned in the previous section) by the *D*_v_ of the vein order and by the number of cells present (*N*), and the BSV and MSV by multiplying the square of half the average bundle or mestome sheath cell diameter (*D*) (as mentioned in the previous section) by the *D*_v_ of the vein order and by π and by the number of cells present (*N*):

(Eqn 12) $$\text{BSSA}=\left(1{}^{\circ}D_{\mathrm{bs}}\times \pi \times 1{}^{\circ}D_{V}\times 1{}^{\circ}N_{\mathrm{bs}}\right)+\left(2{}^{\circ}D_{\mathrm{bs}}\times \pi \times 2{}^{\circ}D_{V}\times 2{}^{\circ}N_{\mathrm{bs}}\right)+\left(3{}^{\circ}D_{\mathrm{bs}}\times \pi \times 3{}^{\circ}D_{V}\times 3{}^{\circ}N_{\mathrm{bs}}\right)$$

(Eqn 13) $$\text{BSPA}=\left(1{}^{\circ}D_{\mathrm{bs}}\times \;1{}^{\circ}D_{V}\times 1{}^{\circ}N_{\mathrm{bs}}\right)+\left(2{}^{\circ}D_{\mathrm{bs}}\times \;2{}^{\circ}D_{V}\times 2{}^{\circ}N_{\mathrm{bs}}\right)+\left(3{}^{\circ}D_{\mathrm{bs}}\times \;3{}^{\circ}D_{V}\times 3{}^{\circ}N_{\mathrm{bs}}\right)$$

(Eqn 14) $$\mathrm{BSV}=\left({\left(1{}^{\circ}D_{\mathrm{bs}}÷2\right)}^{2}\times \;1{}^{\circ}D_{V}\times 1{}^{\circ}N_{\mathrm{bs}}\right)+\left({\left(2{}^{\circ}D_{\mathrm{bs}}÷2\right)}^{2}\times \;2{}^{\circ}D_{V}\times 2{}^{\circ}N_{\mathrm{bs}}\right)+\left({\left(3{}^{\circ}D_{\mathrm{bs}}÷2\right)}^{2}\times \;3{}^{\circ}D_{V}\times 3{}^{\circ}N_{\mathrm{bs}}\right)$$

MSSA, MSPA, and MSV were calculated as in Eqns (Eqn 12), (Eqn 13), (Eqn 14), swapping *D*_bs_ with *D*_ms_ and *N*_bs_ with *N*_ms_, and including the 4° veins.

### Compilation of grass leaf hydraulic and photosynthetic data from the literature

To characterize C_3_ and C_4_ differences in leaf hydraulic and photosynthetic physiology based on the previous literature, we compiled data from published studies after searching for ‘grass’ coupled with ‘leaf physiology’, ‘functional trait’, ‘hydraulics’, and ‘gas exchange’ (Google Scholar and Web of Science). We extracted data for 332 grass species from 37 published studies that included data for the following traits for grasses: light‐saturated leaf photosynthetic rate per leaf area (*A*_area_), stomatal conductance (*g*_s_), leaf hydraulic conductance (*K*_leaf_), leaf xylem conduit hydraulic conductance (*K*_xc_), leaf outside‐xylem hydraulic conductance (*K*_oxc_), vein density (*D*_v_), intrinsic leaf water use efficiency (WUE_i_), leaf water potential at turgor loss point (TLP), leaf water potential at 50% loss of leaf hydraulic conductivity (*P*_50_), and leaf water potential at 80% loss of leaf hydraulic conductivity (*P*_80_) (Table S3). Traits were averaged for species included in several studies. For studies that included data for *K*_leaf_ and *g*_s_ at the species level, we estimated the ratio of *K*_leaf_ : *g*_s_.

### Modeling the native climate of C_3_ and C_4_ grass species

Modeled climate variables were obtained by averaging climate across each species distribution under the assumption that mean phenotypic trait values per species would be indicative of their mean climate variables if gene flow occurs among populations of each species (Sexton *et al*., 2009). Additional details on these methods are provided in a previous publication (Baird *et al*., 2021) and in Methods S8.

### Statistical analyses: phylogenetic comparative methods

We utilized a phylogenetic approach to account for the influence of phylogenetic covariance on average C_3_ and C_4_ trait differences and on trait–trait relationships using the R Language and Environment (R Core Team, 2023). For analyses including the 27 species grown in a common garden, we utilized a previously published time‐calibrated phylogeny (Baird *et al*., 2021). For the compiled grass database, we implemented phylogenetic analyses to test differences in traits between C_3_ and C_4_ species, and to test relationships between traits for all grasses, C_3_ grasses only, and C_4_ grasses only. As each trait in the larger database had a different sample size, we used numerous different phylogenies, depending on the species set, to test for trait differences or trait–trait relationships, each trimmed from a larger global grass phylogeny (Spriggs *et al*., 2014).

Our analyses utilized a custom‐written code that is available on GitHub (https://github.com/smuel‐tylor/grass‐leaf‐size). For analyses of the 27 species from the common garden, and for the 332 species from the compiled database, we examined differences in species‐level trait means between C_3_ and C_4_ species using a phylogenetically corrected analysis of variance (ANOVA), both parametric (based on PGLS) and nonparametric (Garland *et al*., 1993) using the phyloanova package (Revell, 2012). We also tested for relationships between leaf gas exchange and leaf structure, climate, and between leaf hydraulic traits and leaf hydraulic anatomy using phylogenetically corrected regressions, including reduced major axis regressions (PRMA) or generalized least square regressions (PGLS), which incorporate phylogenetic correction as Pagel's λ (Pagel, 1999; Freckleton *et al*., 2002) estimated by maximum likelihood (Pinheiro *et al*., 2019) (Methods S9).

We implemented both phylogenetic and nonphylogenetic tests for analyses of trait–trait relationships across the 332‐species database. The phylogenetic tests resulted in reduced coverage of the trait space and particular C_3_ and C_4_ clades being disproportionately sampled, as many of the phylogenies generated for each trait–trait relationship could not account for all of the species in the database, due to species not being present in the larger phylogeny (Spriggs *et al*., 2014) (see Methods S9 for details). Thus, we present both phylogenetic and nonphylogenetic analyses, but emphasize the nonphylogenetic analyses for our findings on trait–trait relationships for the 332 species. We used the function cor.test to test for significant correlations between traits and present the Pearson correlation coefficient, *r*, and statistical significance *P*‐value. The studies included in the meta‐analysis are provided in Table S3 and the Supporting Information reference list.

### Modeling of hydraulic–stomatal–photosynthetic function of C_3_ and C_4_ species under varying levels of soil and atmospheric drought

We used the mechanistic hydraulic model SurEau v. 2021‐11‐10 (Cochard *et al*., 2021) to simulate the impact of soil drought on the water relations and gas exchange of representative C_3_ and C_4_ plants (Methods S10). We parameterized the photosynthesis model for C_3_ plants (von Caemmerer, 2000, 2021; Osborne & Sack, 2012; Bonan, 2019) and C_4_ (Yin *et al*., 2011; von Caemmerer, 2021), using average values for physiological traits taken from our experiment or others published previously (Table S4). The plant's total hydraulic conductance was adjusted to obtain the operational leaf water potential value for each plant type.

The dependence of stomatal conductance (*g*_s_) and leaf hydraulic conductance (*K*_leaf_) on leaf water potential (Ψ_leaf_) were modeled as:

(Eqn 15) $$g_{s}=\frac{g_{\max}}{1+\exp\left(\frac{\Psi_{\text{leaf}}-\Psi_{\mathrm{gs}50}}{c}\right)}$$

where *g*_max_ is the maximal stomatal conductance, Ψ_leaf_ is the leaf water potential, Ψ_gs50_ is the leaf water potential at 50% stomatal closure, and *c* is a constant, and

(Eqn 16) $$K_{\text{leaf}}=K_{\max}+a\times \Psi_{\text{leaf}}$$

where *K*_max_ is the maximum leaf hydraulic conductance and *a* is the mean slope for *K*_leaf_ vs Ψ_leaf_. For the simulations, we tested hydraulic and photosynthetic responses to declining soil water potential under two VPDs: 0.5 and 3 kPa. Thus, 0.5 kPa VPD was implemented by setting the maximum temperature to 20°C and minimum relative humidity to 78.6%, and the 3 kPa VPD by setting the maximum temperature to 30°C and minimum relative humidity to 29.5%. The simulation starts with soil at field capacity and is allowed to gradually dehydrate under the influence of plant transpiration.

To test the influence of *K*_leaf_ : *g*_s_ on the drought response of gas exchange, in addition to simulating C_3_ and C_4_ grasses, we also simulated a C_3_ grass with the average *K*_leaf_ : *g*_s_ of C_4_ species, and a C_4_ grass with the average *K*_leaf_ : *g*_s_ of C_3_ species (Table S4).

## Results

### Leaf hydraulic transport in grasses and C_4_ hydraulic hyper‐efficiency

**Fig. 3:**
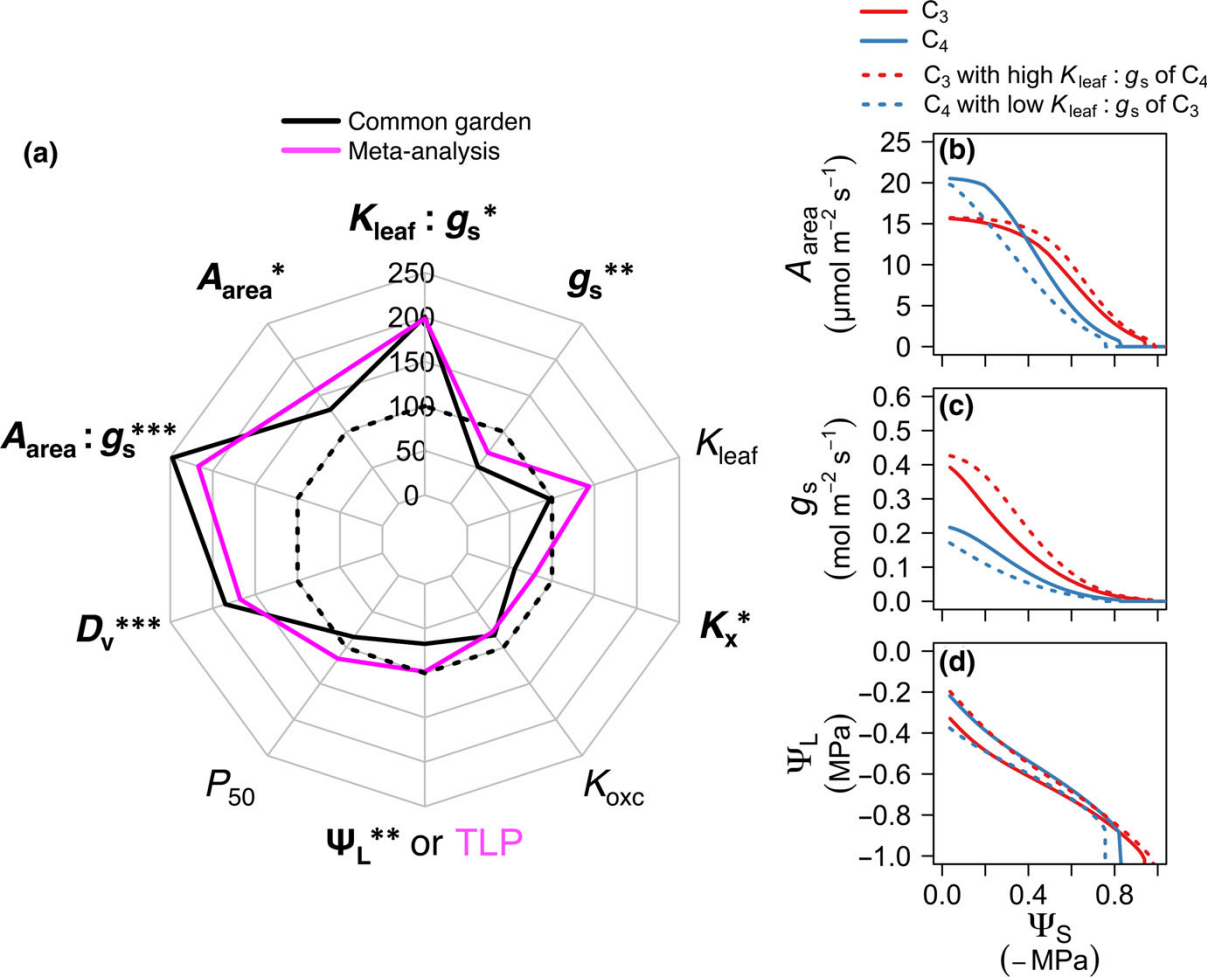
Contrasting hydraulic and photosynthetic physiology of C_3_ and C_4_ grasses and modeled impacts of their traits on physiological declines under drought. Leaf hydraulic and photosynthetic traits including the ratio of leaf hydraulic conductance to stomatal conductance (*K*_leaf_ : *g*_s_), stomatal conductance (*g*_s_), leaf hydraulic conductance (*K*_leaf_), leaf xylem conduit hydraulic conductance (*K*_xc_), leaf outside‐xylem conduit hydraulic conductance (*K*_oxc_), leaf water potential (Ψ_L_), leaf turgor loss point (TLP), leaf water potential at 50% loss of hydraulic conductivity (*P*_50_), leaf vein density (*D*_v_), the ratio of light‐saturated photosynthetic rate per leaf area to stomatal conductance (*A*_area_ : *g*_s_), and light‐saturated photosynthetic rate per leaf area (*A*_area_) for (a) 27 common garden‐grown grasses and 332 grasses from the compiled database, where C_3_ species means were fixed arbitrarily as the 100% reference value (dark dashed line), and the black and magenta solid lines indicate the percent difference between the C_3_ and C_4_ species for the common garden and the meta‐analysis, respectively. Traits in bold type differed significantly between C_3_ and C_4_ species for the 27 phylogenetically matched common garden species (phylogenetic analysis of variance). Modeled responses of (b) *A*_area_, (c) *g*_s_ and (d) Ψ_L_ to declining soil water potential (Ψ_S_) at vapor pressure deficit (VPD) of 0.5 kPa (simulations for 3 kPa VPD in Supporting Information Fig. S2). Significance: *, *P* < 0.05; **, *P* < 0.01; ***, *P* < 0.001, for phylogenetic analysis of variance. Statistics and parameters are found in Tables S2 and S3. Means for *K*_oxc_ excluded *Paspalum diltatum* due to it being an outlier (Dixon's test), though differences in C_3_ and C_4_ were not significant, whether or not this species was included in phylogenetic analysis of variance.

In our meta‐analysis, C_4_ grass species had a 1.4‐fold higher *K*_leaf_ and a twofold higher *K*_leaf_ : *g*_s_ than C_3_ species (Fig. 3a; Tables S2, S3). We also found differences between C_3_ and C_4_ species consistent with our hypotheses and the previous literature (Table 1). In the meta‐analysis, C_4_ grasses had 1.6‐ to 2.2‐fold higher *A*_area_, WUE_i_, and *D*_v_, and 71% lower *g*_s_ (phylogenetic ANOVA; Fig. 3a; Tables S2, S3). Furthermore, in our compiled database, C_3_ and C_4_ grass species were statistically similar in their hydraulic sensitivity to drought, that is, their leaf hydraulic vulnerability to decline of Ψ_leaf_ (*P*_50_ = Ψ_leaf_ at 50% loss of *K*_leaf_) and leaf turgor loss point (TLP = Ψ_leaf_ at turgor loss). In our common garden, *K*_leaf_, *K*_xc_, and *K*_oxc_ did not differ on average between the C_3_ and C_4_ terrestrial species, and the C_4_ species had a twofold higher *K*_leaf_ : *g*_s_ and a higher operating Ψ_leaf_ (Table S2). Notably, phylogenetic and ahistorical tests showed similar results for average C_3_ and C_4_ trait differences and regression analyses (Tables S3, S5).

The importance of high *K*_leaf_ : *g*_s_ in realizing the C_4_ photosynthetic advantage was demonstrated by our integrated whole‐plant modeling of the grass photosynthetic, stomatal, and hydraulic systems (Figs 3b–d, S2). For Ψ_soil_ values representing moist to moderately dry soil, C_4_ species maintained higher modeled Ψ_leaf_ values than C_3_ species, and a superior ability to maintain *g*_s_ and *A*_area_. When simulating a C_4_ grass with the lower *K*_leaf_ : *g*_s_ that was quantified for C_3_ grasses (by reducing *K*_leaf_ proportionately with the lower *g*_s_ of C_4_ species) Ψ_leaf_ and *g*_s_ declined steeply with reduction in Ψ_soil_ and the C_4_ advantage of high *A*_area_ was lost at mild levels of drought. A simulated C_3_ grass with higher *K*_leaf_ : *g*_s_ (i.e. that observed in C_4_ species, achieved by increasing *K*_leaf_) had higher Ψ_leaf_ and moderately higher *g*_s_ or *A*_area_.

### Coordination of hydraulic, stomatal, and photosynthetic function in grasses

**Fig. 4:**
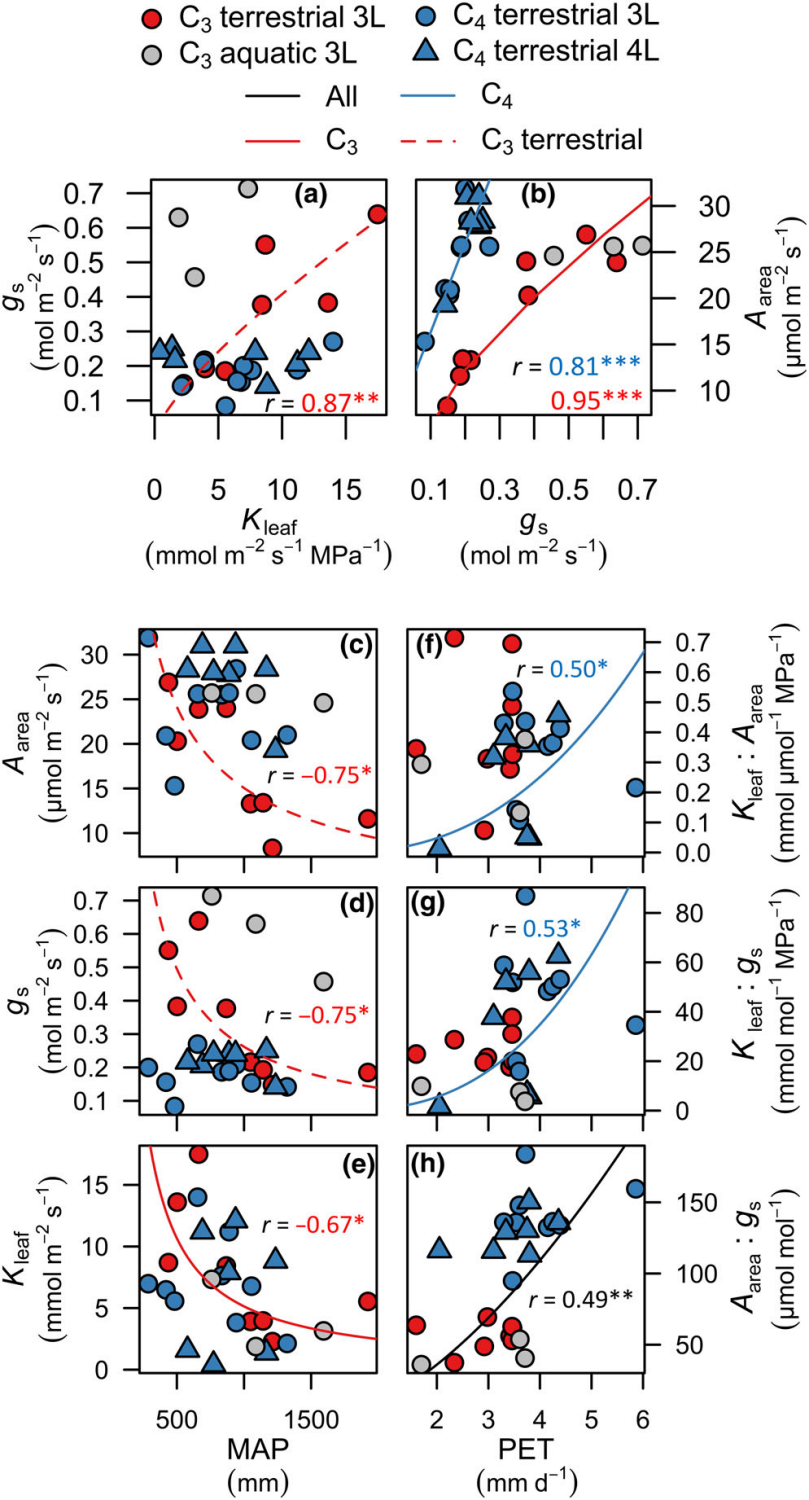
Contrasting physiological trait coordination and adaptation to aridity in C_3_ and C_4_ grasses grown in the common garden. Relationships of (a) stomatal conductance (*g*_s_) with leaf hydraulic conductance (*K*_leaf_), and of (b) light‐saturated leaf photosynthetic rate per leaf area (*A*_area_) with *g*_s_. Relationships of (c) *A*_area_, (d) *g*_s_, and (e) *K*_leaf_ with mean annual precipitation (MAP) for only terrestrial C_3_ plants in (c, d) and all C_3_ in (e), and of (f) the ratio of leaf hydraulic conductance to photosynthetic rate (*K*_leaf_ : *A*_area_) and (g) of the ratio of leaf hydraulic conductance to stomatal conductance (*K*_leaf_ : *g*_s_) to potential evapotranspiration (PET) for C_4_ grasses, and (h) of the ratio of photosynthetic rate to stomatal conductance (*A*_area_ : *g*_s_, i.e. WUE_i_) with PET across all species. Power laws were fitted using phylogenetic reduced major axis regressions (PRMA) for all relationships, except for C_4_ species in (b) in which a linear model better characterized this relationship. Red and blue lines indicate that the relationship was significant across C_3_ or C_4_ species only, respectively, or C_3_ and C_4_ species with varying slopes, as in (b). Only terrestrial species were included for relationships of C_3_ species in (a, c, d). Significance: *, *P* < 0.05; **, *P* < 0.01; ***, *P* < 0.001. *n* = 11 C_3_, 16 C_4_ species. 3L and 4L in the species key refer to the species having three or four longitudinal vein orders, respectively (Fig. 2). Statistics and parameters are found in Supporting Information Tables S6 and S9.

**Fig. 5:**
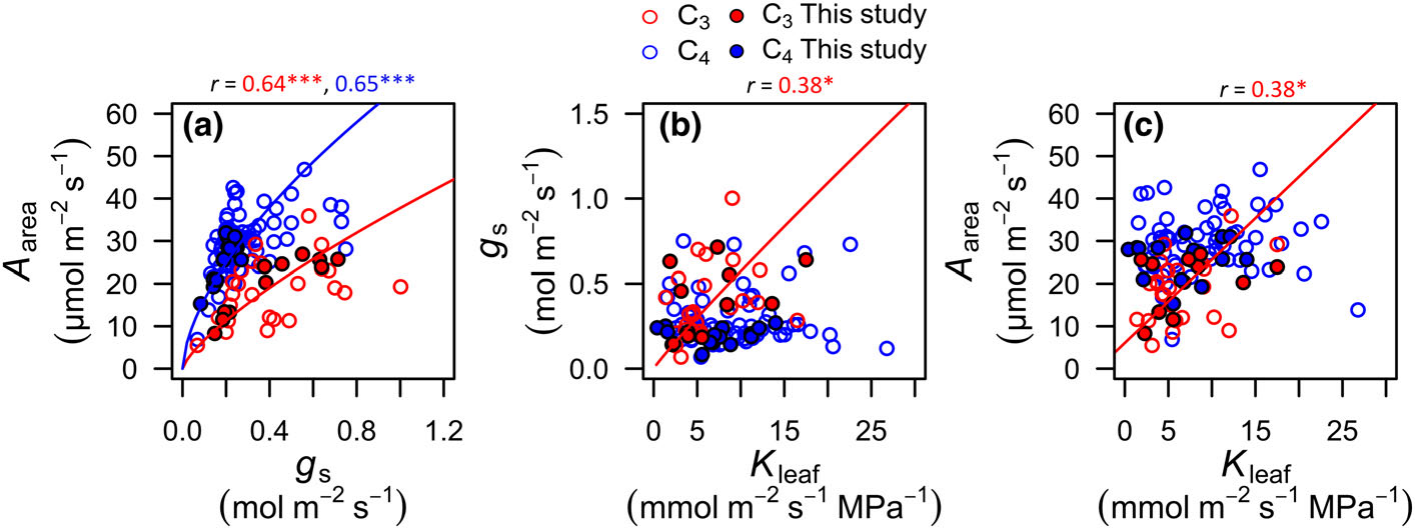
Coordination of leaf physiological traits across grasses, compiled from published studies (Supporting Information Table S3). Relationships of (a) light‐saturated leaf photosynthetic rate (*A*_area_) with stomatal conductance (*g*_s_), (b) *g*_s_ with leaf hydraulic conductance (*K*_leaf_), and (c) *A*_area_ with *K*_leaf_. Lines were fitted with standard major axis (SMA) regressions for log‐transformed data in (a) and (b), and for untransformed data in (c), and drawn when significant: *, *P* < 0.05; **, *P* < 0.01; ***, *P* < 0.001. Values were averaged per species across studies, and analyses included data from this study, represented by closed circles in the plots. Statistics and parameters for both ahistorical and phylogenetic regressions for all pairwise combinations of traits are found in Table S5.

The C_3_ and C_4_ grasses showed contrasting coordination of *K*_leaf_, *g*_s_, and *A*_area_ in both our compiled database and in the 27 species common garden (Figs 4, 5; Tables S5, S6). Among the C_3_ grasses in the common garden, and in the compiled database, *A*_area_ and *g*_s_ scaled with *K*_leaf_ (Figs 4a, 5a; Tables S5, S6). C_4_ grasses showed no association of gas exchange with hydraulic traits, with low *g*_s_ and moderate to high *A*_area_ across the range of *K*_leaf_, relative to C_3_ species (Figs 4a, 5b,c; Table S5). C_3_ and C_4_ species differed in the relationship of *A*_area_ to *g*_s_ (Figs 4b, 5a). Among C_3_ species, while *A*_area_ initially increased with *g*_s_, at high *g*_s_ beyond *c*. 0.4 mol m^−2^ s^−1^ there were slight gains in *A*_area_. Among C_4_ species, there was a steeper relationship, shifted toward a higher *A*_area_ at a given *g*_*s*_ and without evidence of saturation at high *g*_s_.

### Anatomical drivers of grass leaf hydraulic and photosynthetic function

**Fig. 6:**
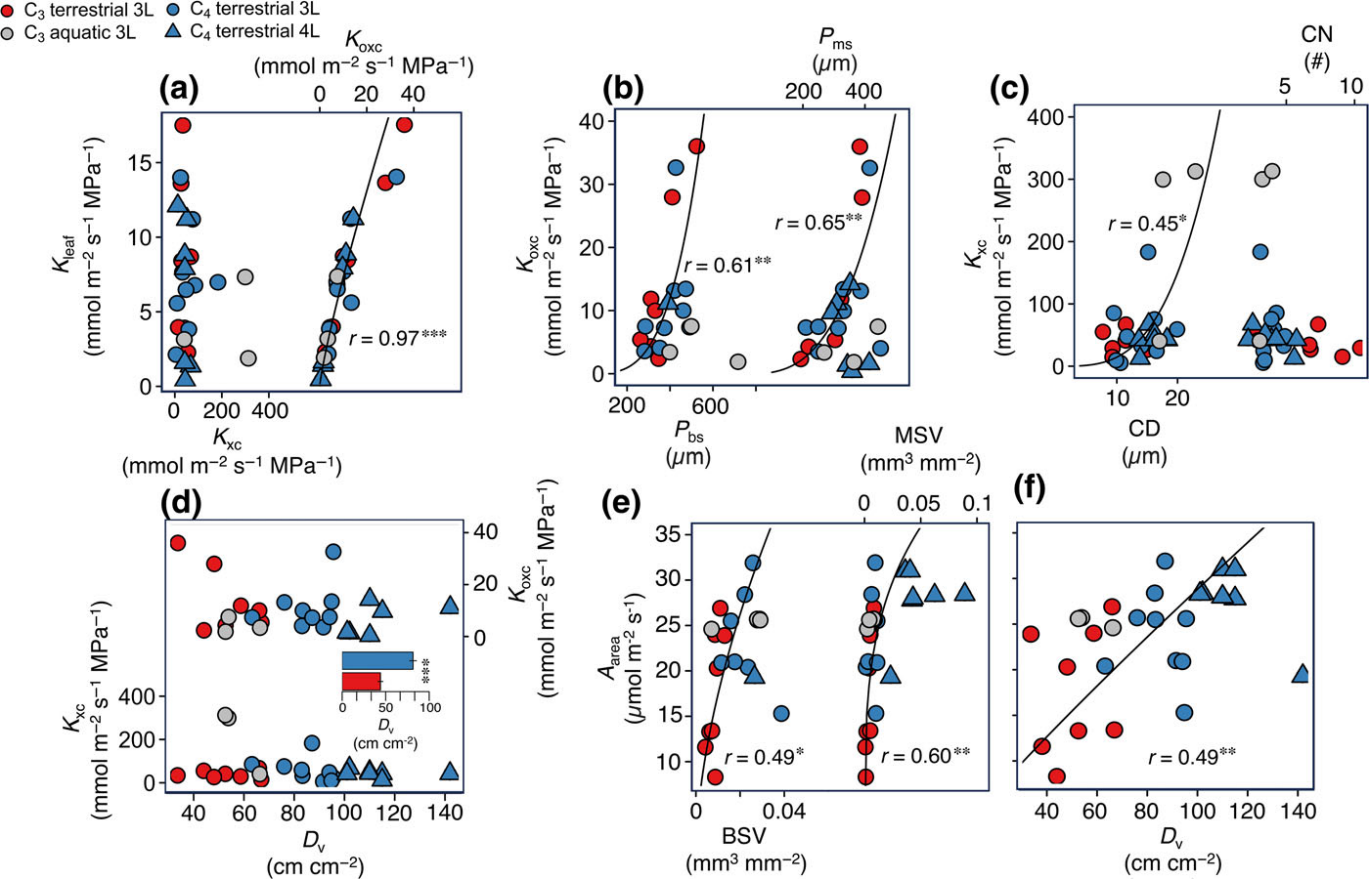
Anatomical drivers of leaf hydraulic and photosynthetic physiology of C_3_ and C_4_ grasses. Across 27 C_3_ and C_4_ grass species grown in a common garden, (a) leaf hydraulic conductance (*K*_leaf_) and leaf xylem conduit lumen hydraulic conductance (*K*_xc_) were statistically independent, and *K*_leaf_ was closely related to leaf outside‐xylem conduit hydraulic conductance (*K*_oxc_). (b) *K*_oxc_ variation was associated with the perimeters of the vein bundle sheath (*P*_bs_) and vein mestome sheath (*P*_ms_) tissues. (c) *K*_xc_ variation was associated with variation in vein conduit diameter (CD) but independent of conduit number (CN). (d) Both *K*_xc_ and *K*_oxc_ were independent of vein density (*D*_v_). (e) A higher light‐saturated photosynthetic rate (*A*_area_) was associated with a higher volume of the bundle sheath (BSV) and mestome sheath (MSV) per leaf area, and (f) *D*_v_. See Supporting Information Table S1 for trait definitions and units. Power laws were fitted using phylogenetic reduced major axis regressions (PRMA) for all relationships across all species, except those in (b) which were significant when considering terrestrial C_3_ species and C_4‐3L_ species together. The species *Paspalum dilatatum* was excluded from plots involving *K*_oxc_ as it was an outlier (Dixon's test), though relationships were significant with or without this species. Significance: *, *P* < 0.05; **, *P* < 0.01; ***, *P* < 0.001. *n* = 11 C_3_, 16 C_4_ species. Statistics and parameters are found in Tables S6 and S7.

We examined the anatomical drivers of grass leaf hydraulic and photosynthetic capacity across the 27 diverse C_3_ and C_4_ common garden‐grown species (Fig. 6; Tables S5, S7, S8). *K*_leaf_, *K*_xc_, and *K*_oxc_ were related to *A*_area_ and vascular anatomy (Tables S6, S7).

Among C_3_ and C_4_ grass species, variation in *K*_leaf_ was independent of *K*_xc_, and related to variation in *K*_oxc_ (Fig. 6a). Among C_3_ and C_4_ species, *K*_oxc_ and *K*_leaf_ increased positively with the outer perimeter of the bundle and mestome sheaths (*P*_bs_ and *P*_ms_; Fig. 6b). Across species, *K*_xc_ was also strongly related to anatomical traits. *K*_xc_ increased positively with the xylem conductances of 1° midvein, and 2° large and 3° intermediate longitudinal vein orders (i.e. *K*_xc‐vein order_; Fig. S3a–c; Table S7), and considering each longitudinal leaf vein order, the vein *K*_xc_ scaled positively with its CD (Fig. S3m–p; Table S8). *K*_xc_ increased with higher CD but was independent of CN averaged across vein orders, and of *D*_v_ (Fig. 6c; Table S7). The higher *D*_v_ of C_4_ species was not associated with an advantage in hydraulic capacity (Fig. 6d; Table S7). Although the higher minor *D*_v_ of C_4_ grasses would contribute to a twofold higher minor vein xylem construction cost (CC_minor_), the reduction in minor vein CN at similar CD offsets that additional cost (whether cell wall thickness is considered as constant as in Eqn 9, or as proportional to CD with an exponent < 1), leading to an overall *K*_xc‐minor_/CC_minor_ similar to that of C_3_ species (Table S2).

Among C_3_ and C_4_ grass species, *A*_area_ was related to venation and sheath traits, including *D*_v_, IVD, vein surface area per leaf area (VSA_total_), vein volume per leaf area (VV_total_), and bundle and mestome sheath volume per leaf area (BSV and MSV; Figs 6e,f, S4; Table S6). Among C_3_ grasses, *A*_area_ was related to the major vein density (*D*_v‐major_) and vein surface area (VSA_major_) (Table S6).

### Adaptation of leaf hydraulics and gas exchange to climate in grasses

C_4_ grasses were native to climates of lower average aridity index (AI) than C_3_ grasses (Table S2). Furthermore, climatic associations of leaf hydraulics and gas exchange differed between C_3_ and C_4_ grasses (Fig. 4). C_3_ grasses native to colder and drier climates, that is, lower MAT and MAP, had higher *A*_area_, *g*_s_, and *K*_leaf_ (Fig. 4c–e; Table S9). By contrast, across C_4_ grasses, *K*_leaf_, *g*_s_, and *A*_area_ were decoupled from MAP, MAT, PET, and AI, but higher *K*_leaf_ : *g*_s_ and *K*_leaf_ : *A*_area_ were associated with environments with higher PET (Fig. 4f,g; Table S9). Across all species, high WUE_i_ was associated with higher PET and AI (Fig. 4h; Table S9).

## Discussion

Our study provides novel evidence of the critical influence of leaf hydraulic anatomy and physiology on photosynthetic function and adaptation to aridity among grasses, highlighting multiple contrasts across levels of organization for C_3_ and C_4_ grasses. The maintenance of C_4_ leaf hydraulic capacity, despite the evolution of lower transpirational demand, leads to a hydraulic surplus and enables stomata to remain open, facilitating the C_4_ photosynthetic advantage. This hydraulic surplus leads to contrasting hydraulic and photosynthetic coordination among C_3_ and C_4_ grasses, resolving paradoxes relating to their vascular anatomy and function, and explains mechanisms of their adaptation to aridity. Our results provide implications for the evolution, ecology, and biogeography of grasses in past and present ecosystems, and applications in agriculture.

The lower *g*_s_ and/or higher *K*_leaf_ of C_4_ grasses leads to a disproportionately higher *K*_leaf_ : *g*_s_ in C_4_ grasses. Our analyses indicate that this higher *K*_leaf_ : *g*_s_ provides hyper‐efficient water transport that is required to achieve higher maximum photosynthetic rates, and enables adaptation to aridity. Hyper‐efficient water transport enables higher operating Ψ_leaf_ during gas exchange, maintaining *g*_s_, and resulting in high *A*_area_ and WUE_i_. The high *K*_leaf_ : *g*_s_ of C_4_ grasses would be essential to prevent stomatal closure that could obviate much of their C_4_ biochemical advantage, as hypothesized previously (Taylor *et al*., 2010, 2011; Osborne & Sack, 2012). The steeper slope for the relationship of *A*_area_ and *g*_s_ among C_4_ grasses is consistent with the C_4_ carbon concentrating mechanism eliminating mesophyll resistance limitations on CO_2_ assimilation (Bjorkman, 1971) (Figs 4b, 5a), and renders the gas exchange of C_4_ species much more sensitive to stomatal closure that would be driven by declining Ψ_leaf_.

We found contrasting associations of *A*_area_ and *g*_s_ with *K*_leaf_ among C_3_ vs C_4_ grasses. For C_3_ grasses, the associations of these variables indicate investment in hydraulic supply to match demand and are consistent with that previously observed for *A*_area_ and *K*_leaf_ among C_3_ grasses, diverse C_3_ plant species and species within the C_3_ lineage *Viburnum* (Brodribb *et al*., 2007; Scoffoni *et al*., 2016; Zhou *et al*., 2021). Yet, the decoupling of *A*_area_ and *g*_s_ from *K*_leaf_ among C_4_ grasses results from the evolution of consistently lower *g*_s_, which would be selected for in the evolution of C_4_. Thus, in C_4_ grasses, the evolution of a disproportionate hydraulic supply to demand (*K*_leaf_ : *g*_s_) leads to decoupling of *A*_area_ and *g*_s_ from *K*_leaf_, as has been previously proposed (Zhou *et al*., 2021).

The determination of leaf hydraulic capacity (*K*_leaf_) by the conductance of the outside‐xylem pathways (*K*_oxc_) can explain paradoxes relating to grass leaf vasculature and hydraulic function. Whereas the higher *D*_v_ of C_4_ grasses could in theory drive a higher *K*_leaf_, the C_4_ grasses had higher *K*_leaf_ only when analyzing the compiled database that included diverse plants grown in different environments, and not in our common garden experiment considering phylogenetically matched species grown in a standardized way. The contrast may thus reflect an influence on the meta‐analysis of plasticity in trait values, for example if C_4_ grasses would tend to have been experimentally grown or measured in sunnier, warmer conditions. Overall, the determination of *K*_leaf_ by *K*_oxc_ rather than *K*_xc_ indicates that a higher *D*_v_ would not drive a higher *K*_leaf_ through higher *K*_xc_ across grasses. A higher minor *D*_v_ did not even drive a higher *K*_xc_, as grasses with higher minor *D*_v_ had narrower minor veins containing fewer xylem conduits (Fig. S5; Table S10). The low constraint by *K*_xc_ on *K*_leaf_ is also consistent with the high efficiency of axial transport through the grass parallel vein architecture relative to radial water transport (Givnish, 1979). A dominant bottleneck in hydraulic transport outside the xylem conduits in grasses is consistent with the low membrane permeability of the bundle sheath (Scoffoni *et al*., 2023), which may be adaptive for equilibration of water potentials across the mesophyll, and to reduce the tension in the xylem, protecting it from embolism (Cochard *et al*., 1994; Stiller *et al*., 2003; Scoffoni *et al*., 2017, 2023) (Fig. S6). The perimeters of the bundle and mestome sheath tissue layers were correlates of *K*_oxc_, highlighting the importance of transport through sheath cell walls thought to be typically highly resistant and hydrophobic and especially through membrane aquaporins and/or plasmodesmata (Sade *et al*., 2015; Ohtsuka *et al*., 2018). Notably, our measurements of *K*_leaf_ and thus of *K*_oxc_ are defined for the water pathway ending in the mesophyll protoplasts (Scoffoni *et al*., 2023); our findings of *K*_oxc_ being low and dynamic suggest resistant membranes and/or cell walls between the xylem and mesophyll cells. Such a highly resistant membrane and cell wall would be consistent with a recent study reporting airspace subsaturation in C_4_ grasses in association with high resistance in the cell membranes and/or cell walls adjacent to the leaf intercellular airspaces (Márquez *et al*., 2024). In that study, cell membrane resistance was considered a key mechanism enabling stomata to remain hydrated and open and thus avoid a steep decline of *A*_area_ in C_4_ species. That mechanism would operate in parallel with the high *K*_leaf_ : *g*_s_ of C_4_ species shown in this study, which would enable leaf water potential to remain high enough to avoid driving stomatal closure. The important role of *K*_oxc_ in constraining *K*_leaf_ highlights the need to develop direct measurements of its determinants, for example membrane conductivity.

With respect to xylem conductance, the influence of CD on *K*_xc_ within and across grass species is consistent with a key role for the large variation in conduit diameters across species, especially the large conduits in the major longitudinal veins (Fig. 2a–c), which accounted for the bulk of *K*_xc_ (> 98% across species) (Figs S3, S6; Table S2). Our findings suggest very limited constraints by the cost of xylem on the evolution of high *D*_v_ for C_4_ carbon concentration in grasses. Despite the higher minor *D*_v_ of C_4_ grasses, given their lower minor vein CN and similar CD to C_3_ species, we found that C_3_ and C_4_ species had similar minor vein xylem hydraulic conductance relative to minor vein xylem construction cost (*K*_xc‐minor_/CC_minor_) (Table S2). Our finding for grass leaves thus contrasts with the finding that stems of C_4_ eudicots evolved lower hydraulic conductance associated with reduced xylem construction costs (Kocacinar & Sage, 2003, 2004). Shifts in xylem properties may also be linked with mechanical properties that contribute to herbivory resistance and/or optimizing light‐use efficiency (Duarte *et al*., 2023).

The associations between *A*_area_ and numerous vein and sheath traits provide new mechanistic insights. The influence of high *D*_v_ on *A*_area_ was not mediated directly by hydraulics, as *K*_leaf_, *K*_xc_, and *K*_oxc_ were not associated with *D*_v_, *D*_v‐major_, or VSA_major_. This finding was consistent with *K*_leaf_ depending most strongly on high outside‐xylem limitation (Fig. 6a). Positive associations between *A*_area_ and *D*_v_, *D*_v‐major_, and VSA_major_ may be related to the transport of sugar rather than water, as higher vascularity would reduce transport resistance between veins and mesophyll and provide greater vein sheath surface for exchange, and more parallel transport pathways (Adams *et al*., 2013). However, across the C_3_ species, the relationship of *A*_area_ with vein sheath traits BSV and MSV may be consistent with a hydraulic basis, arising because the associations of *K*_oxc_ and *K*_leaf_ with *P*_bs_ and *P*_ms_ enable higher *g*_s_ and *A*_area_ (Tables S6, S7). By contrast, among C_4_ grasses, the positive association between *A*_area_ and BSV and MSV was not mediated by *K*_oxc_ or *K*_leaf_, and arises from contributions to greater volumes of photosynthetic vein sheath tissue (Figs 1, 6; Table S6) (Christin *et al*., 2013), and because the higher *D*_v‐minor_ of C_4_ species increased BSV and MSV (Table S2).

The higher *K*_leaf_ : *g*_s_ in C_4_ grasses and the contrasting coordination of leaf hydraulics and gas exchange for C_3_ and C_4_ grasses indicate differential mechanisms for adaptation to macroclimate. Our simulations show that a high *K*_leaf_ : *g*_s_ is as necessary as their C_4_ biochemistry in providing the photosynthetic advantage of C_4_ over C_3_ grasses under even mild drought, and therefore is vital to their domination of open, lower rainfall environments (Edwards & Smith, 2010) (Fig. 4g). Notably, a high *K*_leaf_ : *g*_s_ would have been critical for C_4_ species to maintain open stomata under the low CO_2_ atmosphere experienced during the proliferation of the C_4_ grass lineages in the Miocene, and to sustain the high *A*_area_ that fueled their competitive advantage (Edwards *et al*., 2010), especially in dry climates, and would also potentially support leaf transpirational cooling in hot environments (Blonder *et al*., 2023). As C_4_ arose repeatedly in grass evolution, along with lower *g*_s_ driven by the development of fewer and smaller stomata, a high *K*_leaf_ : *g*_s_ would have evolved repeatedly during the adaptation of high *D*_v_ coupled with C_4_ vein sheath traits in dry and sunny environments (Sage, 2004; Osborne & Sack, 2012; Taylor *et al*., 2012; Christin *et al*., 2013; Zhou & Osborne, 2024). Thus, high *K*_leaf_ : *g*_s_ would have evolved as a precursor adaptation or simultaneously with C_4_ biochemistry (Marazzi *et al*., 2012), and should be considered as a critical target in engineering novel C_4_ crop species.

The diversification of C_3_ grasses with higher *A*_area_, *g*_s_, and *K*_leaf_ under cold and dry climates is consistent with stress avoidance by capitalizing on short rainfall pulses and growing seasons to compensate for reduced performance during dry and cold periods (Grubb, 1998; Liu *et al*., 2019). The differential associations of hydraulic and photosynthetic traits with climate in C_3_ and C_4_ grasses would contribute to their avoidance of drought, that is, their compensating for climatic aridity with rapid growth during the shorter duration of high moisture (Fig. 4). The similar average *P*_50_ and TLP of C_3_ and C_4_ species is also consistent with their adaptation to competitive growth when water is available, and adaptation to aridity typically achieved through drought avoidance (Fig. 3a). Notably, adaptation to climate in our study was resolved by testing annual mean macroclimate variables from species' native ranges, which were strongly associated with seasonal mean variables (see Methods S8). We note that species would adapt differently both to seasonality and to different axes of aridity, for example soil and atmospheric drought and their interactions. Indeed, the adaptive trait mechanisms shown here may also be associated with other aspects of ecology, including herbivore susceptibility and flammability, and further disentangling this complexity for trait–climate associations forms a major avenue for future research.

## Competing interests

None declared.

## Author contributions

ASB, SHT, CPO and LS conceptualized the project, developed the methodology, and validated the data. ASB, SHT, JP‐K, CV, YZ, TW, HC, CS, EJE, CPO and LS performed the data curation, and reviewed and edited the manuscript. ASB and LS undertook the formal analyses and wrote the original draft. ASB, CS, CPO and LS acquired the funding. ASB, SHT, JP‐K, TW, CS, EJE, CPO and LS performed the investigations. ASB, SHT, JP‐K, CPO and LS administered and supervised the project. ASB, SHT, JP‐K, TW, CS, CPO and LS provided the resources. ASB, SHT and HC wrote the software. ASB and CV provided the data visualization.

## Disclaimer

The New Phytologist Foundation remains neutral with regard to jurisdictional claims in maps and in any institutional affiliations.

## Supporting information


**Fig. S1** Phylogenetic tree and biogeographic distributions of 27 grass species grown in a common garden and sampled for hydraulic and anatomical traits.
**Fig. S2** Results of simulation modeling of the hydraulic‐stomatal‐photosynthetic system of C_3_ and C_4_ grasses.
**Fig. S3** Coordination of leaf photosynthetic rate with leaf hydraulic anatomy.
**Fig. S4** Testing determinants of leaf xylem conduit hydraulic conductance (*K*_xc_).
**Fig. S5** Relationships of 3° leaf hydraulic conductance and vein traits in C_4_ grasses.
**Fig. S6** Partitioning of the leaf hydraulic resistance and leaf xylem conductance across vein orders.
**Methods S1** Plant growth conditions.
**Methods S2** Preparation of leaf transverse cross sections.
**Methods S3** Quantification of leaf hydraulic conductance.
**Methods S4** Quantification of leaf gas exchange.
**Methods S5** Vein order categorization for anatomy measurements.
**Methods S6** Details on the calculation of leaf xylem conduit hydraulic conductance.
**Methods S7** Quantification of additional potential correlates of leaf outside‐xylem conduit hydraulic conductance.
**Methods S8** Modeling the native climate of C_3_ and C_4_ grasses.
**Methods S9** Details on functions and approaches used for statistical analyses.
**Methods S10** Modeling of hydraulic‐stomatal‐photosynthetic function of C_3_ and C_4_ species during drought and varying vapor pressure deficit.

**Table S1** Variables quantified for C_3_ and C_4_ grass species: leaf hydraulic physiology, gas exchange physiology, venation and structure and vein sheath anatomy.
**Table S2** Species of grasses (Poaceae) included in the common garden study, subfamily, tribe, C_3_/C_4_ photosynthetic pathway, C_4_ subtype, seed source, accession number, seed treatment for germination, terrestrial/aquatic, sun/shade, and mean, ±SE of anatomical and morphological traits measured and climate data, and statistics from phylogenetic analysis of variance below trait means.
**Table S3** Hydraulic, photosynthetic and anatomical data for 332 grass species from published studies and used to test relationships of leaf gas exchange and hydraulics across species, and to test average differences between C_3_ and C_4_ species.
**Table S4** Model parameters used to test the importance of high ratio of leaf hydraulic conductance to stomatal conductance (*K*_leaf_ : *g*_s_) for C_4_ photosynthetic advantage in wet and drying soil.
**Table S5** Correlation matrices for trait–trait relationships for the 332 grass species database.
**Table S6** Statistics and parameters for associations of leaf photosynthetic traits with leaf hydraulic and anatomical traits across all species, terrestrial species only, C_3_ species only, C_3_ terrestrial species only and C_4_ species only, from the common garden.
**Table S7** Statistics and parameters for associations of leaf hydraulic traits with leaf hydraulic, photosynthetic and anatomical traits across all species, terrestrial species only, C_3_ species only, C_3_ terrestrial species only and C_4_ species only, from the common garden.
**Table S8** Statistics and parameters for associations of leaf xylem hydraulic conductance per vein order with leaf hydraulic anatomy across all species from the common garden.
**Table S9** Statistics and parameters for associations of climate with leaf hydraulic, photosynthetic and anatomical traits across all species, terrestrial species only, C_3_ species only, C_3_ terrestrial species only and C_4_ species only, from the common garden.
**Table S10** Statistics and parameters for coordination or trade‐offs of leaf structural traits across all species from the common garden.
Please note: Wiley is not responsible for the content or functionality of any Supporting Information supplied by the authors. Any queries (other than missing material) should be directed to the *New Phytologist* Central Office.

## Data Availability

All data are available in Tables S2–S4. Code used for phylogenetic analyses was previously published (Baird *et al*., 2021) and is available on GitHub (https://github.com/smuel‐tylor/grass‐leaf‐size).
