# Supplementary material for: Resolving the contrasting leaf hydraulic adaptation of C3 and C4 grasses
Source: New Phytol. 2025 Jan 5;245(5):1924–39. doi: 10.1111/nph.20341 (PMC11798900; doi:10.1111/nph.20341)
Supplement: Supplementary file 1 — Fig. S1 Phylogenetic tree and biogeographic distributions of 27 grass species grown in a common garden and sampled for hydraulic and anatomical traits. Fig. S2 Results of simulation modeling of the hydraulic‐stomatal‐photosynthetic system of C3 and C4 grasses. Fig. S3 Coordination of leaf photosynthetic rate with leaf hydraulic anatomy. Fig. S4 Testing determinants of leaf xylem conduit hydraulic conductance (K xc). Fig. S5 Relationships of 3° leaf hydraulic conductance and vein traits in C4 grasses. Fig. S6 Partitioning of the leaf hydraulic resistance and leaf xylem conductance across vein orders. Methods S1 Plant growth conditions. Methods S2 Preparation of leaf transverse cross sections. Methods S3 Quantification of leaf hydraulic conductance. Methods S4 Quantification of leaf gas exchange. Methods S5 Vein order categorization for anatomy measurements. Methods S6 Details on the calculation of leaf xylem conduit hydraulic conductance. Methods S7 Quantification of additional potential correlates of leaf outside‐xylem conduit hydraulic conductance. Methods S8 Modeling the native climate of C3 and C4 grasses. Methods S9 Details on functions and approaches used for statistical analyses. Methods S10 Modeling of hydraulic‐stomatal‐photosynthetic function of C3 and C4 species during drought and varying vapor pressure deficit. [file NPH-245-1924-s001.docx]

## New Phytologist Supporting Information

Article title: Resolving the contrasting leaf hydraulic adaptation of C_3_ and C_4_ grasses

Authors: Alec S. Baird, Samuel H. Taylor, Jessica Pasquet-Kok, Christine Vuong, Yu Zhang, Teera Watcharamongkol, Hervé Cochard, Christine Scoffoni, Erika J. Edwards, Colin P. Osborne, Lawren Sack

Article acceptance date: 27 November 2024

The following Supporting Information is available for this article:

Fig. S1 Phylogenetic tree and biogeographic distributions of 27 grass species grown in a common garden and sampled for hydraulic and anatomical traits.

**Fig. S2** Results of simulation modeling of the hydraulic-stomatal-photosynthetic system of C_3_ and C_4_ grasses.

**Fig. S3** Coordination of leaf photosynthetic rate with leaf hydraulic anatomy.

**Fig. S4** Testing determinants of leaf xylem conduit hydraulic conductance (*K*_xc_).

**Fig. S5** Relationships of 3° leaf hydraulic conductance and vein traits in C_4_ grasses.

**Fig. S6** Partitioning of the leaf hydraulic resistance and leaf xylem conductance across vein orders.

**Table S1** Variables quantified for C_3_ and C_4_ grass species: leaf hydraulic physiology, gas exchange physiology, venation and structure and vein sheath anatomy.

**Table S2** Species of grasses (Poaceae) included in the common garden study, subfamily, tribe, C_3_/C_4_ photosynthetic pathway, C_4_ subtype, seed source, accession number, seed treatment for germination, terrestrial/aquatic, sun/shade, and mean, ± standard errors of anatomical and morphological traits measured and climate data, and statistics from phylogenetic analysis of variance below trait means.

**Table S3** Hydraulic, photosynthetic and anatomical data for 332 grass species from published studies and used to test relationships of leaf gas exchange and hydraulics across species, and to test average differences between C_3_ and C_4_ species.

**Table S4** Model parameters used to test the importance of high ratio of leaf hydraulic conductance to stomatal conductance (*K*_leaf_/*g*_s_) for C_4_ photosynthetic advantage in wet and drying soil.

**Table S5** Correlation matrices for trait-trait relationships for the 332 grass species database.

**Table S6** Statistics and parameters for associations of leaf photosynthetic traits with leaf hydraulic and anatomical traits across all species, terrestrial species only, C_3_ species only, C_3_ terrestrial species only and C_4_ species only, from the common garden.

**Table S7** Statistics and parameters for associations of leaf hydraulic traits with leaf hydraulic, photosynthetic and anatomical traits across all species, terrestrial species only, C_3_ species only, C_3_ terrestrial species only and C_4_ species only, from the common garden.

**Table S8** Statistics and parameters for associations of leaf xylem hydraulic conductance per vein order with leaf hydraulic anatomy across all species from the common garden.

**Table S9** Statistics and parameters for associations of climate with leaf hydraulic, photosynthetic and anatomical traits across all species, terrestrial species only, C_3_ species only, C_3_ terrestrial species only and C_4_ species only, from the common garden.

**Table S10** Statistics and parameters for coordination or trade-offs of leaf structural traits across all species from the common garden.

**Methods S1** Plant growth conditions

**Methods S2** Preparation of leaf transverse cross sections

**Methods S3** Quantification of leaf hydraulic conductance

**Methods S4** Quantification of leaf gas exchange

**Methods S5** Vein order categorization for anatomy measurements

**Methods S6** Details on the calculation of leaf xylem conduit hydraulic conductance

**Methods S7** Quantification of additional potential correlates of leaf outside-xylem conduit hydraulic conductance

**Methods S8** Modeling the native climate of C_3_ and C_4_ grasses

**Methods S9** Details on functions and approaches used for statistical analyses

**Methods S10** Modeling of hydraulic-stomatal-photosynthetic function of C_3_ and C_4_ species during drought and varying vapor pressure deficit

Fig. S1 Phylogenetic tree and biogeographic distributions of 27 grass species grown in a common garden and sampled for hydraulic and anatomical traits.

(a) black branches, 11 C_3_ species; light blue branches, 9 C_4-3L_ species; dark blue branches, 7 C_4-4L_ species (Baird *et al.*, 2021). Map of the distributions of (b) 11 C_3_ and (c) 16 C_4_ species (Baird *et al.*, 2021). Note the five sister C_3_ groups within the PACMAD.

**Fig. S2** Results of simulation modeling of the hydraulic-stomatal-photosynthetic system of C_3_ and C_4_ grasses.

The responses of (a) light-saturated leaf net photosynthetic rate (*A*_area_) (b) stomatal conductance (*g*_s_) and (c) leaf water potential (Ψ_L_) to declining soil water potential (Ψ_S_) at high vapor pressure deficit (VPD) (3 kPa).

**Fig. S3** Testing determinants of leaf xylem conduit hydraulic conductance (*K*_xc_).

Relationships of whole leaf xylem conduit hydraulic conductance (*K*_xc_) with (a) midvein xylem conduit hydraulic conductance (*K*_xc-midvein_), (b) second order xylem conduit hydraulic conductance (*K*_xc-large_), (c) third order xylem conduit hydraulic conductance (*K*_xc-intermediate_) and (d) fourth order xylem conduit hydraulic conductance (*K*_xc-small_). Independence of *K*_xc_ from (e) midvein vein length per area (*D*_v-midvein_), (f) second order vein length per area (*D*_v-large_), (g) third order vein length per area (*D*_v-intermdiate_) and (h) fourth order vein length per area (*D*_v-small_). Independence of vein order specific *K*_xc_ from (i) *D*_v-midvein_ (j) *D*_v-large_, (k) *D*_v-intermediate_ and (l) *D*_v-small_, and relationships with vein order specific conduit diameter (*CD*) for (m) first order midvein (*CD*_midvein_), (n) second order large veins (*CD*_large_), (o) third order intermediate veins (*CD*_intermediate_) and (p) fourth order small veins (*CD*_small_), and independence from vein order specific conduit number (*CN*) for (q) midvein conduit number (*CN*_midvein_), (r) second order conduit number (*CN_l_*_arge_), (s) third order conduit number (*CN*_intermediate_) and (t) fourth order conduit number (*CN*_small_). Lines were fitted with phylogenetic reduced major axis regressions (PRMA) and drawn when significant: **P* < 0.05; ***P* < 0.01; ****P* < 0.001. *N* = 10 C_3_, 16 C_4_ species. Statistics and parameters are found in Tables S7 and S8.

**Fig. S4** Coordination of leaf photosynthetic rate with leaf hydraulic anatomy.

Relationships of light-saturated leaf photosynthetic rate (*A*_area_) with (a) total vein density (*D*_v_), (b) interveinal-distance (*IVD*), (c) total vein surface area per area (*VSA*), (d) total vein volume per area (VVA), (e) total bundle sheath surface area per area (*BSSA*), (f) total mestome sheath surface area per area (*MSSA*), (g) total bundle sheath volume per area (*BSV*) and (h), total mestome sheath volume per area (*MSV*). Lines were fitted with phylogenetic reduced major axis regressions (PRMA) and drawn when significant: **P* < 0.05; ***P* < 0.01; ****P* < 0.001. *N* = 11 C_3_, 16 C_4_ species. Statistics and parameters are found in Table S6.

**Fig. S5** Relationships of 3° leaf hydraulic conductance and vein traits in C_4_ grasses.

Independence of 3° vein leaf hydraulic conductance (*K*_leaf_) from (a) 3° vein density (*D*_v_) (the main contributor to higher *D*_v_ in C_4_ plants) and (c) from 3° vein conduit diameter (CD). Relationships of 3° leaf vein diameter (*VD*) with (b) 3° *D*_v_ and (d) 3° conduit number (CN). Lines were fitted with phylogenetic reduced major axis regressions (PRMA) and drawn when significant: **P* < 0.05; ***P* < 0.01; ****P* < 0.001. *N =* 11 C_3_, 16 C_4_. Statistics and parameters are found in Table S10.

**Fig. S6** Partitioning of the leaf hydraulic resistance and leaf xylem conductance across vein orders

(a) Leaf hydraulic resistance (*R*_leaf_) of the outside-xylem and xylem pathways. (b) Leaf xylem conduit hydraulic conductance (*K*_xc_) of each longitudinal vein order. (c) Percentage of *R*_leaf_ of the outside-xylem and xylem pathways. (d) Percentage of *K*_xc_ of each longitudinal vein order. *Paspalum dilatatum* was excluded from outside-xylem partitions in (a) and (c) because it was an outlier (Dixon’s outlier test)

**Methods S1** Plant growth conditions

Seeds were acquired from seed banks and commercial sources (Table S2), and prior to germination were surface-sterilized with 10% NaClO and 0.1% Triton X-100 detergent, rinsed three times with sterile water, and sown on plates of 0.8% agar sealed with Micropore surgical tape (3M, St. Paul, MN). Seeds were germinated in chambers maintained at 26˚C, under moderate intensity cool white fluorescent lighting with a 12 hour photoperiod. When roots ranged from 2-3 cm long, seedlings were transplanted to 3.6 L pots with potting soil (1:1:1.5:1.5:3 of coarse vermiculite: perlite: washed plater sand: sandy loam: peat moss). We included aquatic species to capture high phylogenetic and ecological diversity and selection under different environmental pressures.

Plants were grown in a common-garden at the UCLA Plant Growth Center (minimum, mean and maximum daily values for temperature: 20.1, 23.4 and 34.0^o^C; for relative humidity: 28, 50 and 65%; and mean and maximum photosynthetically active radiation during daylight period: 107 and 1988 µmol photons m^-2^ s^-1^; HOBO Micro Station with Smart Sensors; Onset, Bourne, MA). The mean photosynthetically active radiation was averaged from 4 am to 10:30 pm. Plants were arranged in six randomized blocks spread over three benches, with one individual per species per block (*n* = 6 except: *Alloteropsis semialata*, *n* = 4) and two blocks per bench. Plants were irrigated daily with water containing fertilizer (200-250 ppm of 20:20:20 N:P:K; Scotts Peters Professional water soluble fertilizer; Everris International B.V., Geldermalsen, The Netherlands).

Common garden designs typically better resolve adaptation than measuring different species in contrasting native conditions, by reducing plastic trait expression that can strongly affect interspecific comparisons (Cordell *et al*., 1998; Dunbar-Co *et al*., 2009; Givnish & Montgomery, 2014; Scoffoni *et al*., 2015; Huxman *et al*., 2022). We recognize that a common garden may introduce uncertainty for certain conclusions regarding interspecific comparisons, especially when the common environment varies strongly from the climate of some species’ native ranges, as traits would thus be influenced by the climate transfer distance (i.e., the difference in climate between the common growth conditions and the species’ native range). In this study, the common garden was designed to reflect mesic conditions with ample nutrients and water supply. One study of diverse species found that species’ divergences from across-species trait-climate relationships (with traits measured in a common-garden and climate variables representing the species native ranges) correlated with their climate transfer distances; thus, a strong trait-climate relationship would imply a reduced importance of climate transfer distance in influencing species-differences in common-garden measured traits (Medeiros *et al*., 2023). Overall, the trait-climate relationships observed in our study would support the adaptation of traits with respect to climate, irrespective of plasticity (Cordell *et al*., 1998; Dunbar-Co *et al*., 2009; Givnish & Montgomery, 2014; Scoffoni *et al*., 2015; Huxman *et al*., 2022).

**Methods S2** Preparation of leaf transverse cross sections

At the center of the leaf, rectangular samples were cut and under vacuum over the duration of one week, infiltrated with low viscosity acrylic resin (L.R. White; London Resin Co., UK). Infiltrated samples were then set in resin in gelatin capsules to dry at 55 C overnight. From these samples, transverse cross sections of 1 um thickness and of varying width (species dependent) were then prepared using glass knives (LKB 7800 KnifeMaker;LKB Produkter; Bromma, Sweden) in a rotary microtome (Leica Ultracut E, Reichert-Jung California, USA), placed on slides and stained with 0.01% toluidine blue in 1% sodium borate (w/v). Slides were imaged with a 5×, 20×, and 40× objective using a light microscope (Leica Lietz DMRB; Leica Microsystems) and camera with imaging software (SPOT Imaging Solution; Diagnostic Instruments, Sterling Heights, Michigan USA).

**Methods S3** Quantification of leaf hydraulic conductance

We measured the leaf hydraulic conductance (*K*_leaf_) between 9 Feb and 25 June 2010 using the steady-state evaporative flux method (EFM) (Sack & Scoffoni, 2012). Measurements were typically made for 2-3 leaves per plant from 6 plants, resulting in 6-18 leaves per species.

Stems were cut from the plant with a fresh razor blade under water in the growth center, placed in a polythene bag (Whirl-Pak; Nasco, Fort Atkinson, WI, USA), and transported to the lab for measurement. Individual grass leaves were wrapped in parafilm around a plastic rod of appropriate diameter (3-18 mm; McMasterCarr, Elmhurst, IL), re-cut with a fresh razor blade under distilled water and rapidly connected to tubing with a compression fitting (Omnifit A2227 bore adaptor; Omnifit, Cambridge, UK and 18 mm diameter compression coupling Dynamax, Houstin, TX). The tubing contained distilled water that was degassed for at least 8 h with a vacuum pump (GAST Manufacturing, Inc, Michigan, USA), and refiltered 0.2μm; Syringe filter, Cole-Parmer, Vernon Hills, IL) and connected the leaf to a cylinder of water on a balance (Mettler Toledo, XS205 DualRange, ±0.01/0.1 mg), which logged data every 30 s to a computer for the calculation of flow rate into the petiole (*E*). Leaves were held adaxial surface upwards using a wood frame strung with fishing line, which held the leaf horizontal and immobile above a large box fan (Lakewood Engineering & Manufacturing Company, Chicago, Illinois, USA). Leaves were illuminated by a light source (model 73828 1000 W, “UV filter”; Sears, Roebuck, Hoffman Estates, Illinois, USA) suspended above a Pyrex glass container (Corning Incorporated, Corning, New York, USA) filled with water above the leaf producing >1000 µmol m^-^² s^-1^ PAR at the leaf surface. Leaf temperature was maintained between 23-28°C during the experiment. Leaves were allowed to transpire on the fan apparatus for at least 30 min, until the flow rate stabilized with a coefficient of variation < 5% for at least 5 min. A 30 min period was chosen to ensure that leaves had sufficient time to acclimate to light, which previous studies have shown to enhance *K*_leaf_ by several-fold for certain species(Sack *et al.*, 2002; Tyree *et al.*, 2005; Cochard *et al.*, 2007; Scoffoni *et al.*, 2008). When flow rate was very low (< 8 µg s^-1^) and did not stabilize with that criterion, the measurement was continued until a running average of the last ten flow measurements stabilized with a coefficient of variation <5%. Additionally, flow rate was plotted against time to ensure stability. Measurements were discontinued if the flow suddenly changed, either due to leakage in the system or apparent blockage by particles or air bubbles. Leaf temperature was recorded with a thermocouple thermometer (Cole-Parmer Instrument Company, Vernon Hills, Illinois, USA) and the final 5 min of flow rate were averaged. The leaf was quickly removed from the tubing, the cut end dabbed dry, and the leaf sealed into a Whirlpak bag, which had been exhaled into. Following at least 20 min equilibration, the final leaf water potential (Ψ_f_) was measured using a pressure chamber (Plant Moisture Stress, Model 1000, Albany, Oregon, USA). To correct for changes in *K*_leaf_ induced by the temperature dependence of water viscosity, *K*_leaf_ values were standardized to 25 °C (Weast, 1974; Sack *et al.*, 2003). Measurements were made for 2-3 leaves per plant for each of 6 plants (except 9 plants for *A. ternipes*, 4 plants for *A. semialata*, and for *L. sorghoidea* 5 and 8 leaves were measured from two plants); overall 6-18 leaves per species were measured. We removed outliers for each species using Dixon’s outlier test (Sokal & Rohlf, 1995); up to 0-3 outliers for 14 of the 28 species; data for 6-18 leaves remained, 12 on average. The values for *K*_leaf_ with and without removing outliers were highly correlated across species (*r* = 0.96; *P* < 0.001), and all the findings of the study were robust to whether or not outliers were maintained in the dataset.

**Methods S4** Quantification of leaf gas exchange

Steady state leaf gas exchange was measured using a LI-6400 XT portable photosynthesis system (LI-COR, Lincoln, Nebraska, USA). The leaf chamber was maintained at 25°C, with reference CO_2_ 400 ppm, and PPFD 2000 µmol m^-2^ s^-1^, which was assumed to be saturating irradiance for these species (Taylor *et al*., 2010). The relative humidity was 60-80%, leading to vapor pressure deficits (VPD) of 0.80-1.6 kPa (overall mean 1.1 kPa). Measurements were made for 1-2 leaves from each of six plants (except from five plants for *A. purpurea*, four plants for *A. semialata*, seven plants for *P. australis*, and for *L. sorghoidea* three leaves from each of two plants). Overall, 5-9 leaves per species were measured, with 6 on average. Leaf-area normalized values were determined for stomatal conductance (*g*_s_) and net photosynthetic rate per leaf area (*A*_area_). The ratio of intercellular to ambient CO_2_ (*C*_i_/*C*_a_) was also estimated, since it is negatively related to water use efficiency. Leaves were harvested, scanned for leaf area (Canon Scan Lide 90, Canon USA, Lake Success, NY), dried at 70°C for at least 48h and weighed to determine the leaf dry mass per unit area (LMA) and net CO_2_ assimilation rate per unit leaf dry mass (*A*_mass_).

The physiological data collection was spread over a 4-month period, though we note that across this period greenhouse plants generally experienced >6 hours of high sunlight daily. Measurements were staggered to standardize measurements of the diverse species grown in the greenhouse common garden for a common plant growth stage. All species were set out for germination at the same time, and planted into the experiment when the hypocotyl emerged, and measurements were conducted when plants had grown many leaves of which >3 were large and mature. We thus aimed to control for developmental stage, given the variation across the diverse species in their growth rates and senescence within our common garden.

**Methods S5** Vein order categorization for anatomy measurements

Vein orders were established for each species based on species-specific phylogenetic history (Christin *et al.*, 2013; Lundgren *et al.*, 2019), and by estimations of vein size, presence/absence of enlarged metaxylem, and presence/absence of fibrous tissue above and/or below the vein (Ellis, 1976; Evert, 2006; Baird *et al.*, 2021). We categorized major veins as the 1° vein, i.e., the midvein, the large central vein containing the largest xylem and fibrous tissue, and 2°, or “large” veins smaller than the midvein and of similar structure (Evert, 2006; Baird *et al.*, 2021). We categorized minor veins as the 3° or “intermediate”, 4° or “small” veins, and 5° or “transverse” veins (Evert, 2006; Baird *et al.*, 2021). For C_3_ grasses and most C_4_ grasses, the smallest visible longitudinal veins were 3° “intermediate” veins. In NADP-ME C_4_ grasses of the subfamily Panicoideae, 4° small veins evolved, which co-opted their mestome sheath for carbon reduction(Christin *et al.*, 2013); these species thus have both 3° “intermediate” and 4° “small” vein orders (*Alloteropsis semialata, Andropogon gerardii, Cenchrus setaceus, Digitaria ciliaris, Digitaria eriantha, Echinochloa crus-galli, Paspalum dilatatum*), whereas Panicoideae species that co-opted the outer bundle sheath for carbon reduction (*Alloteropsis cimicina, Chloris elata, Chloris gayana, Eragrostis cilianensis, Eriachne aristidea, Panicum virgatum*) or non-Panicoideae species that co-opted the inner bundle sheath for carbon reduction (*Aristida purpurea, Aristida ternipes, Stipagrotis zeyheri*) lack 4° “small” vein orders. As we lacked cross sections of second-order veins for *Laciacis sorghoidea*, we did not measure its second-order conduit dimensions, and this species was excluded from analyses involving these traits.

**Methods S6** Details on the calculation of leaf xylem conduit hydraulic conductance

The assumption of additive resistances of vein orders in an integrated network was validated for eudicotyledonous leaves (Sack *et al*., 2004; Cochard *et al*., 2004). The assumption of additive vein conductances in grasses was developed based on the vein orders being arranged in parallel and supplied from the leaf base, and has been applied in experiments partitioning the vein and outside vein conductances (Ocheltree *et al*., 2013, Xiong *et al*., 2017), and validated by the matching of measured *K*_xc_ with *K*_xc_ estimated on the basis of xylem anatomy (Xiong *et al*., 2017). While transverse veins connect the parallel veins, leading to some uncertainty in this partitioning, their effects on partitioning would likely be negligible, as they typically contain a single, extremely small xylem conduit (Botha, 2013*;* Drobnitch *et al*., 2024).

In the calculation of *K*_xc_, the theoretical xylem conductivity (*K*_t_) was first calculated, using Poiseuille’s Equation from conduit dimensions (mmol m s^-1^ MPa^-1^) and converted this value into a xylem conductance in the same units as leaf hydraulic conductance (*K*_leaf_; mmol m^-2^ s^-1^ MPa^-1^), using *K*_xc_ (for a given vein order) = *K*_t_ ×number of veins / (0.5 *LL* × *LA*), where *LL* is leaf length and *LA* is leaf area. This normalization, developed by analogy to normalizing wood conductivity to give a hydraulic conductance per leaf area, assumes that half the water would leave the xylem halfway along the leaf. *K*_xc_ calculated for each longitudinal vein order are summed. Because *D*_v_ = number of veins × *LL* / *LA*, a mathematically equivalent calculation of *K*_xc_ using vein density (*D*_v_, mm mm^-2^) is: *K*_xc_ (for a given vein order) = *K*_t_ × *D*_v_ (for that vein order)/0.5 × *LL*^2^.

Our estimation of *K*_xc_ would be somewhat larger than a flow-derived axial hydraulic conductance because some resistances are not accounted for, such as extra-conduit resistances (including end-walls and the cell walls water crosses on exiting the xylem). We assigned the symbol “*K*_xc_” to this variable, for the theoretical conductance of the xylem conduits, rather than “*K*_x_” to emphasize that this does not include extra-conduit resistance, which would be part of the “outside-xylem conduit conductance” (*K*_oxc_). These extra-conduit resistances would lower actual axial hydraulic conductance. For wood, vessel end resistance tends to scale with vessel lumen resistance (Sperry *et al.*, 2005), explaining the correlation of *K*_xc_ with measured axial xylem conductance per leaf area. Further, in a study of rice varieties, a flow-derived *K*_xc_ was strongly correlated with *K*_xc_ measured from anatomy as in this study(Xiong *et al.*, 2017).

Pit resistance between xylem conduits through which water moves axially and that of pits through which water moves radially upon leaving the xylem would be captured by *K*_oxc_. Yet, previous work has demonstrated that such resistance tends to be correlated with axial xylem resistance and of a similar magnitude, such that it would account for a small part of outside xylem conduit resistance (*R*_oxc_, i.e., 1/*K*_oxc_) (Lazzarin *et al.*, 2016; Olson *et al.*, 2021).

The estimation of *K*_oxc_ on the basis of anatomy (subtracting *K*_xc_ from *K*_leaf_) follows previous studies of grasses and nongrasses (Sack *et al*., 2004, Ocheltree *et al*., 2013, Scoffoni *et al*., 2016; Xiong *et al*., 2017*;* Xiong & Nadal, 2020); this estimation is indirect, and similar in approach to other physiological decomposition analyses whereby an unknown is derived from measurements, such as unit leaf rate (or net assimilation rate) derivation in plant growth analyses from relative growth rate and leaf area ratio (Lambers & Oliveira, 2019) or of intercellular carbon concentration from gas exchange measurements of stomatal conductance and photosynthetic rate (Farquhar *et al.,* 1980; *Zhou et al*., 2019). A previous study among rice cultivars found that *K*_xc_ measured on the basis of anatomy matched *K*_xc_ quantified physiologically (Xiong *et al.,* 2017).

**Methods S7** Quantification of additional potential correlates of leaf outside-xylem conduit hydraulic conductance

We quantified interveinal-distance (*IVD*) and the maximum distance from veins to stomata (*D*_m_) (Brodribb *et al.*, 2010). We quantified *IVD* by measuring the distance between two adjacent 3° veins, excluding the bundle and mestome sheath cells. *D*_m_ can be estimated as the hypotenuse of the triangle formed from *IVD* and the distance from the minor veins to stomata. We estimated *D*_m_ as:

$D_{m}=(\sqrt{\left( IVD \right)^{2}+\left( D_{\mathrm{adaxial}} \right)}+\sqrt{\left( IVD \right)^{2}+\left( D_{\mathrm{abaxial}} \right)})\div2$ (17)

where *IVD* is interveinal distance, and *D*_adaxial_ is the vertical distance from the vein sheath to the adaxial epidermis stomata and *D*_abaxial_ is the distance from the vein sheath to the abaxial epidermis stomata.

**Methods S8** Modeling the native climate of C_3_ and C_4_ grasses

We extracted biogeographical records from the Global Biodiversity Information Facility web portal (https://www.gbif.org) for each C_3_ and C_4_ species in the common garden. For each location per species, values for mean annual temperature (*MAT*) and mean annual precipitation (*MAP*) were extracted from WorldClim2 5-arch minute resolution (Fick & Hijmans, 2017), and values for aridity index (*AI*) and potential evapotranspiration (*PET*) were extracted from CRU TS4.01 01 (Harris *et al.*, 2014). Values were averaged across each location per species per climate variable. Adaptation to macroclimatic aridity is complex. We included modelled climate variables aridity index (AI), potential evapotranspiration (PET), mean annual precipitation (MAP) and mean annual temperature (MAT) to represent overall macroclimate variation among the diverse species. Notably, in a previous paper, the leaf and vein traits of diverse grasses were strongly related to both mean annual and growing season climate variables (Baird *et al*., 2021). To test the robustness of annual macroclimatic variables, we examined relationships across species of MAT and MAP with growing season temperature and precipitation (GST and GSP) respectively. We estimated growing season variables considering growing season months as those with mean temperature ≥ 4 °C and precipitation ≥ 2× the mean monthly temperature (Lasky *et al*., 2012), and calculated growing season length as the number of months that fulfilled these criteria, and GST and GSP respectively by averaging the mean temperatures and summing the mean precipitation of these months (Lasky *et al*., 2012). As these relationships between annual and seasonal mean climate variables were highly significant for both raw and log-transformed data (*r* = 0.91-0.98, *p* < 0.001), our findings of trait relationships with mean annual climate variables also reflect adaptation to the growing season.

**Methods S9** Details on functions and approaches used for statistical analyses

Our analytical approach used both phylogenetically explicit statistics and nonphylogenetic (ahistorical) statistics for greatest insight, depending on which dataset was being analyzed. For analyses of the 27 common garden grown species, we implemented phylogenetically explicit tests. Our use of phylogenetic statistics provides conservative test for adaptative hypotheses, as associations would be supported only when they are generalizable across independent divergence events. Notably, phylogenetic niche conservatism (PNC) is set aside; according to PNC, clades with particular associations of traits and climate would occupy particular niches and ongoing selection keeps them there, and thus associations of traits and climate would not necessarily arise from many independent divergences. Additionally, phylogenetic comparative analysis may not account properly for singular events in evolution (Uyeda *et al*., 2018). For the common garden experiment, our species selection sampled multiple lineages with independent transitions between C_3_ and C_4_, and also sampled broadly across the different grass subclades, and our use of phylogenetic analyses focused on repeated divergences to rigorously test for adaptive associations is appropriate to this design. Notably, our previous work implementing both phylogenetic and ahistorical tests for this species set showed similar associations (Baird *et al*., 2021). For the meta-analysis of the 332 species, we implemented both phylogenetically explicit and ahistorical statistics. By contrast with the 27 common garden grown species, we were unable to approximate an even representation of the different grass lineages in this meta-analysis, and thus statistics calculated with or without phylogenetic covariance would be more likely to diverge. Further, phylogenetic regression analyses for this dataset resulted in smaller sample sizes because many species were not represented in the phylogenetic tree, potentially reducing the trait and taxon space explored. Second, the putative similarity of the associations calculated with and without phylogenetic covariance would provide insight into important axes of functional variation across scales, including global trends associated with adaptive divergence among major lineages, and trends generalized among lineages of closely related species. While we present both phylogenetically explicit and ahistorical statistics for the meta-analysis, we emphasize the results for the ahistorical statistics, given the greater species representation.

For phylogenetic reduced major axis (PRMA) regressions we used the function phyl.RMA (Revell, 2012) and for phylogenetic generalized least square (PGLS) regressions we used the function corPagel (Revell, 2012) in combination with gls (Pinheiro *et al*., 2019) and optimized (Paradis & Schliep, 2019) to establish maximum likelihood estimates of λ. Utilization of PRMA or PGLS depended on the two traits being tested. The least squares approach is preferred when a dependent *y*-variable is related to an independent *x*-variable, when (1) there is less error in natural variation and/or measurement error in *x* than *y* and/or in cases when (2) *y* is determined by or to be predicted from *x*, but never *x* from *y* (Poorter & Sack, 2012; Sack *et al.*, 2012; Baird *et al.*, 2021)*.* The reduced major axis approach is preferable in cases in which (1) *x* and *y* have similar error and/or when (2) *x* and *y* are codetermined, or their relationship is due to an underlying functional coordination or could be predicted using each other (Poorter & Sack, 2012; Sack *et al.*, 2012; Baird *et al.*, 2021). We note that only the slope and intercept vary between PRMA and PGLS, and a significant relationship under either PRMA or PGLS is meaningful. We examined trait relationships for log-transformed variables.

**Methods S10** Modeling of hydraulic-stomatal-photosynthetic function of C_3_ and C_4_ species during drought and varying vapor pressure deficit

The model SurEau has been previously used to demonstrate the role of stomatal closure rates in drought resistance (Martin-StPaul *et al.*, 2017), the impact of leaf hydraulic vulnerability on gas exchange in model species (Scoffoni *et al.*, 2018) the importance of leaf minimum epidermal conductance on plant water relations (Duursma *et al.*, 2019) and to predict tree mortality under drought and high temperatures (Brodribb *et al.*, 2019; Cochard, 2021), and has thus been integral for addressing fundamental questions in plant physiology, especially under a changing climate. SurEau simulates water flow and hydraulic pressure gradients based on the principles of thermodynamics, with parameterization of internal and external hydraulic resistances and capacitances in the plant and soil. A detailed description of the model and its function, including equation derivations and code, is provided by (Cochard *et al.*, 2021).

**Supporting Information References**

**Baird AS, Taylor SH, Pasquet-Kok J, Vuong C, Zhang Y, Watcharamongkol T, Scoffoni C, Edwards EJ, Christin P-A, Osborne CP, *et al.*** **2021**. Developmental and biophysical determinants of grass leaf size worldwide. *Nature* **592**: 242–247.

**Batool H**. **2021**. The effect of age and drought on the recovery of midday leaf hydraulics and physiological traits in oat (*Avena nuda*). *International Journal of Agriculture and Biology* **25**: 581–590.

**Bellasio C, Quirk J, Beerling DJ**. **2018**. Stomatal and non-stomatal limitations in savanna trees and C_4_ grasses grown at low, ambient and high atmospheric CO_2_. *Plant Science* **274**: 181–192.

**Bellasio C, Stuart-Williams H, Farquhar GD, Flexas J**. **2023**. C_4_ maize and sorghum are more sensitive to rapid dehydration than C_3_ wheat and sunflower. *New Phytologist* **240**: 2239–2252.

**Botha CEJ**. **2013**. A tale of two neglected systems–structure and function of the thin- and thick- walled sieve tubes in moncotyledonous leaves. *Frontiers in Plant Science* **4**

**Brodribb TJ, Cochard H, Dominguez CR**. **2019**. Measuring the pulse of trees; using the vascular system to predict tree mortality in the 21st century. *Conservation Physiology* **7**: coz046.

**Brodribb TJ, Feild TS, Sack L**. **2010**. Viewing leaf structure and evolution from a hydraulic perspective. *Functional Plant Biology* **37**: 488–498.

**Brodribb TJ, Holbrook NM**. **2004**. Stomatal protection against hydraulic failure: a comparison of coexisting ferns and angiosperms. *New Phytologist* **162**: 663–670.

**Cano FJ, Sharwood RE, Cousins AB, Ghannoum O**. **2019**. The role of leaf width and conductances to CO_2_ in determining water use efficiency in C_4_ grasses. *New Phytologist* **223**: 1280–1295.

**Christin P-A, Osborne CP, Chatelet DS, Columbus JT, Besnard G, Hodkinson TR, Garrison LM, Vorontsova MS, Edwards EJ**. **2013**. Anatomical enablers and the evolution of C_4_ photosynthesis in grasses. *Proceedings of the National Academy of Sciences* **110**: 1381–1386.

**Cochard H**. **2021**. A new mechanism for tree mortality due to drought and heatwaves. *Peer Community Journal* **1**.

**Cochard H, Nardini A, Coll L**. **2004**. Hydraulic architecture of leaf blades: where is the main resistance? *Plant, Cell & Environment* **27**: 1257–1267.

**Cochard H, Pimont F, Ruffault J, Martin-StPaul N**. **2021**. SurEau: a mechanistic model of plant water relations under extreme drought. *Annals of Forest Science* **78**: 1–23.

**Cochard H, Venisse J-S, Barigah TS, Brunel N, Herbette S, Guilliot A, Tyree MT, Sakr S**. **2007**. Putative role of aquaporins in variable hydraulic conductance of leaves in response to light. *Plant Physiology* **143**: 122–133.

**Cordell S, Goldstein G, Mueller-Dombois D, Webb D, Vitousek PM**. **1998**. Physiological and morphological variation in *Metrosideros polymorpha*, a dominant Hawaiian tree species, along an altitudinal gradient: the role of phenotypic plasticity. *Oecologia* **113**: 188–196.

**Crookston RK, Moss DN**. **1974**. Interveinal distance for carbohydrate transport in leaves of C_3_ and C_4_ Grasses1. *Crop Science* **14**: cropsci1974.0011183X001400010038x.

**Drobnitch ST, Kray JA, Gleason SM, Ocheltree TW**. **2024**. Comparative venation costs of monocotyldon and dicotyledon species in the eastern Colorado steppe. *Planta* **260**:2

**Dunbar-Co S, Sporck MJ, Sack L**. **2009**. Leaf trait diversification and design in seven rare taxa of the Hawaiian *Plantago* radiation. *International Journal of Plant Sciences* **170**: 61–75.

**Duursma RA, Blackman CJ, Lopéz R, Martin-StPaul NK, Cochard H, Medlyn BE**. **2019**. On the minimum leaf conductance: its role in models of plant water use, and ecological and environmental controls. *New Phytologist* **221**: 693–705.

**Ellis RP**. **1976**. A procedure for standardizing comparative leaf anatomy in the Poaceae. I. The leaf-blade as viewed in transverse section. *Bothalia* **12**: 65–109.

**Evert RF**. **2006**. *Esau’s Plant Anatomy: Meristems, Cells, and Tissues of the Plant Body: Their Structure, Function, and Development*. John Wiley.

**Farquhar GD, von Caemmerer S, Berry JA**. **1980**. A biochemical model of photosynthetic CO_2_ assimilation in leaves of C_3_ plants. *Planta* **149**: 78–90.

**Fick SE, Hijmans RJ. 2017.** WorldClim 2: new 1-km spatial resolution climate surfaces for global land areas. *International Journal of Climatology*. **37**: 4302–4315.

**Givnish T, Montgomery RA**. **2014**. Common-garden studies on adaptive radiation of photosynthetic physiology among Hawaiian lobeliads. *Proceedings of the Royal Society B* **281**: 20132944

**Griffin-Nolan RJ, Chieppa J, Knapp AK, Nielsen UN, Tissue DT**. **2023**. Coordination of hydraulic and morphological traits across dominant grasses in eastern Australia. *Functional Ecology* **37**: 1126–1139.

**Griffin-Nolan RJ, Ocheltree TW, Mueller KE, Blumenthal DM, Kray JA, Knapp AK**. **2019**. Extending the osmometer method for assessing drought tolerance in herbaceous species. *Oecologia* **189**: 353–363.

**Harris I, Jones PD, Osborn TJ, Lister DH. 2014.** Updated high-resolution grids of monthly climatic observations – the CRU TS3.10 dataset. *International Journal of Climatology*. **34**: 623–642

**Hattersley PW**. **1984**. Characterization of C_4_ type leaf anatomy in grasses (Poaceae). mesophyll: bundle sheath area ratios. *Annals of Botany* **53**: 163–180.

**Holloway-Phillips M-M, Brodribb TJ**. **2011**. Minimum hydraulic safety leads to maximum water-use efficiency in a forage grass. *Plant, Cell & Environment* **34**: 302–313.

**Hoover DL, Koriakin K, Albrigtsen J, Ocheltree T**. **2019**. Comparing water-related plant functional traits among dominant grasses of the Colorado Plateau: Implications for drought resistance. *Plant and Soil* **441**: 207–218.

**Huxman TE, Winkler DE, Mooney KA**. **2022**. A common garden super-experiment: An impossible dream to inspire possible synthesis. *Journal of Ecology* **110**: 997–1004.

**Israel WK, Watson-Lazowski A, Chen Z-H, Ghannoum O**. **2022**. High intrinsic water use efficiency is underpinned by high stomatal aperture and guard cell potassium flux in C_3_ and C_4_ grasses grown at glacial CO_2_ and low light. *Journal of Experimental Botany* **73**: 1546–1565.

**Jacob V, Choat B, Churchill AC, Zhang H, Barton CVM, Krishnananthaselvan A, Post AK, Power SA, Medlyn BE, Tissue DT**. **2022**. High safety margins to drought-induced hydraulic failure found in five pasture grasses. *Plant, Cell & Environment* **45**: 1631–1646.

**Kawamitsu Y, Hakoyama S, Agata W, Takeda T**. **1985**. Leaf interveinal distances corresponding to anatomical types in grasses. *Plant and Cell Physiology* **26**: 589–593.

**Lambers H, Oliveira RS**. **2019**. *Plant Physiological Ecology*. Springer Cham

**Lasky JR, Des Marais DL, McKay JK, Richards JM, Juenger TE, Keitt TH**. **2012**. Characterizing genomic variation of *Arabidopsis thaliana*: the roles of geography and climate. *Molecular Ecology* **21**: 5512–5529.

**Lazzarin M, Crivellaro A, Williams CB, Dawson TE, Mozzi G, Anfodillo T**. **2016**. Tracheid and pit anatomy vary in tandem in a tall *Sequoadendron giganteum* tree. *IAWA Journal* **37**: 172–185.

**Liu H, Taylor SH, Xu Q, Lin Y, Hou H, Wu G, Ye Q**. **2019**. Life history is a key factor explaining functional trait diversity among subtropical grasses, and its influence differs between C_3_ and C_4_ species. *Journal of Experimental Botany* **70**: 1567–1580.

**Lundgren MR, Dunning LT, Olofsson JK, Moreno-Villena JJ, Bouvier JW, Sage TL, Khoshravesh R, Sultmanis S, Stata M, Ripley BS, *et al.*** **2019**. C_4_ anatomy can evolve via a single developmental change. *Ecology Letters* **22**: 302–312.

**Májeková M, Hájek T, Albert ÁJ, de Bello F, Doležal J, Götzenberger L, Janeček Š, Lepš J, Liancourt P, Mudrák O**. **2021**. Weak coordination between leaf drought tolerance and proxy traits in herbaceous plants. *Functional Ecology* **35**: 1299–1311.

**Martin-StPaul N, Delzon S, Cochard H**. **2017**. Plant resistance to drought depends on timely stomatal closure. *Ecology Letters* **20**: 1437–1447.

**Martre P, Durand J-L, Cochard H**. **2000**. Changes in axial hydraulic conductivity along elongating leaf blades in relation to xylem maturation in tall fescue. *The New Phytologist* **146**: 235–247.

**Medeiros CD, Henry C, Trueba S, Anghel I, Díaz de León Guerrero SD, Pivovaroff A, Fletcher LR, John GP, Lutz JA, Mendez Alonzo R, Sack L. 2023**. Predicting plant species climate preferences on the basis of mechanistic traits. *Functional Ecology* **37**: 2786–2808.

**Morgan JA, Brown RH**. **1979**. Photosynthesis in grass species differing in carbon dioxide fixation pathways: II. A search for species with intermediate gas exchange and anatomical characteristics 1. *Plant Physiology* **64**: 257–262.

**Ocheltree T, Gleason S, Cao K-F, Jiang G-F**. **2020**. Loss and recovery of leaf hydraulic conductance: Root pressure, embolism, and extra-xylary resistance. *Journal of Plant Hydraulics* **7**: e-001.

**Ocheltree T, Nippert JB, Kirkham MB, Prasad PVV**. **2013**. Partitioning hydraulic resistance in *Sorghum bicolor* leaves reveals unique correlations with stomatal conductance during drought. *Functional Plant Biology* **41**: 25–36.

**Ocheltree TW, Nippert JB, Prasad PVV**. **2014**. Stomatal responses to changes in vapor pressure deficit reflect tissue-specific differences in hydraulic conductance. *Plant, Cell & Environment* **37**: 132–139.

**Ocheltree TW, Nippert JB, Prasad PVV**. **2016**. A safety vs efficiency trade-off identified in the hydraulic pathway of grass leaves is decoupled from photosynthesis, stomatal conductance and precipitation. *New Phytologist* **210**: 97–107.

**Olson ME, Anfodillo T, Gleason SM, McCulloh KA**. **2021**. Tip-to-base xylem conduit widening as an adaptation: causes, consequences, and empirical priorities. *New Phytologist* **229**: 1877–1893.

**Pan L, George-Jaeggli B, Borrell A, Jordan D, Koller F, Al-Salman Y, Ghannoum O, Cano FJ**. **2022**. Coordination of stomata and vein patterns with leaf width underpins water-use efficiency in a C_4_ crop. *Plant, Cell & Environment* **45**: 1612–1630.

**Paradis E, Schliep K. 2019.** ape 5.0: an environment for modern phylogenetics and evolution in R. *Bioinformatics* **35**: 526-528.

**Pathare VS, Koteyeva N, Cousins AB**. **2020a**. Increased adaxial stomatal density is associated with greater mesophyll surface area exposed to intercellular air spaces and mesophyll conductance in diverse C_4_ grasses. *New Phytologist* **225**: 169–182.

**Pathare VS, Sonawane BV, Koteyeva N, Cousins AB**. **2020b**. C_4_ grasses adapted to low precipitation habitats show traits related to greater mesophyll conductance and lower leaf hydraulic conductance. *Plant, Cell & Environment* **43**: 1897–1910.

**Pinheiro J, Bates D, Debroy S, Sarkar D**. **2019**. nlme: linear and nonlinear mixed effect models: R package version 3.1-140, https://CRAN.R-project.org/package=nlme.

**Pinto H, Sharwood RE, Tissue DT, Ghannoum O**. **2014**. Photosynthesis of C_3_, C_3_–C_4_, and C_4_ grasses at glacial CO_2_. *Journal of Experimental Botany* **65**: 3669–3681.

**Poorter H, Sack L. 2012**. Pitfalls and possibilities in the analysis of biomass patterns in plants. *Frontiers in Plant Science*. **3**: 259

**Quirk J, Bellasio C, Johnson DA, Osborne CP, Beerling DJ**. **2019**. C_4_ savanna grasses fail to maintain assimilation in drying soil under low CO_2_ compared with C_3_ trees despite lower leaf water demand. *Functional Ecology* **33**: 388–398.

**Revell LJ**. **2012**. phytools: an R package for phylogenetic comparative biology (and other things). *Methods in Ecology and Evolution* **3**: 217–223.

**Sack L, Cowan PD, Jaikumar N, Holbrook NM**. **2003**. The ‘hydrology’ of leaves: co-ordination of structure and function in temperate woody species. *Plant, Cell & Environment* **26**: 1343–1356.

**Sack L, Melcher PJ, Zwieniecki MA, Holbrook NM**. **2002**. The hydraulic conductance of the angiosperm leaf lamina: a comparison of three measurement methods. *Journal of Experimental Botany* **53**: 2177–2184.

**Sack L, Scoffoni C**. **2012**. Measurement of leaf hydraulic conductance and stomatal conductance and their responses to irradiance and dehydration using the evaporative flux method (EFM). *Journal of Visualized Experiments.* 4179.

**Sack L, Scoffoni C, McKown AD, Frole K, Rawls M, Havran JC, Tran H, Tran T. 2012**. Developmentally based scaling of leaf venation architecture explains global ecological patterns. *Nature Communications* **3**, 837.

**Sack L, Streeter CM, Holbrook NM**. **2004**. Hydraulic analysis of water flow through leaves of sugar maple and red oak. *Plant Physiology* **134**: 1824–1833.

**Saha S, Holbrook NM, Montti L, Goldstein G, Cardinot GK**. **2009**. Water relations of *Chusquea ramosissima* and *Merostachys claussenii* in Iguazu National Park, Argentina. *Plant Physiology* **149**: 1992–1999.

**dos Santos CM, Endres L, da Silva ACS, Silva JV, de Souza Barbosa GV, Froehlich A, Teixeira MM**. **2019**. Water relations and osmolite accumulation related to sugarcane yield under drought stress in a tropical climate. *International Journal of Plant Production* **13**: 227–239.

**Scoffoni C, Albuquerque C, Cochard H, Buckley TN, Fletcher LR, Caringella MA, Bartlett M, Brodersen CR, Jansen S, McElrone AJ, *et al.*** **2018**. The causes of leaf hydraulic vulnerability and its influence on gas exchange in *Arabidopsis thaliana*. *Plant Physiology* **178**: 1584–1601.

**Scoffoni C, Chatelet DS, Pasquet-Kok J, Rawls M, Donoghue MJ, Edwards EJ, Sack L**. **2016**. Hydraulic basis for the evolution of photosynthetic productivity. *Nature Plants* 16072.

**Scoffoni C, Kunkle J, Pasquet-Kok J, Vuong C, Patel A, Montgomery R, Givnish TJ, Sack L**. **2015**. Light-induced plasticity in leaf hydraulics, venation, anatomy and gas exchange in ecologically diverse Hawaiian lobeliads. *New Phytologist*. **207**: 43–58.

**Scoffoni C, Pou A, Aasamaa K, Sack L**. **2008**. The rapid light response of leaf hydraulic conductance: new evidence from two experimental methods. *Plant, Cell & Environment* **31**: 1803–1812.

**Sokal RR, Rohlf FJ**. **1995**. *Biometry*. W.H. Freeman.

**Sperry JS, Hacke UG, Wheeler JK**. **2005**. Comparative analysis of end wall resistivity in xylem conduits. *Plant, Cell & Environment* **28**: 456–465.

**Stiller V, Lafitte HR, Sperry JS**. **2003**. Hydraulic properties of rice and the response of gas exchange to water stress. *Plant Physiology* **132**: 1698–1706.

**Taylor SH, Aspinwall MJ, Blackman CJ, Choat B, Tissue DT, Ghannoum O**. **2018**. CO_2_ availability influences hydraulic function of C_3_ and C_4_ grass leaves. *Journal of Experimental Botany* **69**: 2731–2741.

**Taylor SH, Hulme SP, Rees M, Ripley BS, Ian Woodward F, Osborne CP**. **2010**. Ecophysiological traits in C_3_ and C_4_ grasses: a phylogenetically controlled screening experiment. *New Phytologist* **185**: 780–791.

**Tyree MT, Nardini A, Salleo S, Sack L, El Omari B**. **2005**. The dependence of leaf hydraulic conductance on irradiance during HPFM measurements: any role for stomatal response? *Journal of Experimental Botany* **56**: 737–744.

**Uyeda JC, Zenil-Ferguson R, Pennell MW**. **2018**. Rethinking phylogenetic comparative methods. *Systematic Biology*. **67**: 1091–1109

**Wang X, Du T, Huang J, Peng S, Xiong D**. **2018**. Leaf hydraulic vulnerability triggers the decline in stomatal and mesophyll conductance during drought in rice. *Journal of Experimental Botany* **69**: 4033–4045.

**Weast RC**. **1974**. *Handbook of Chemistry and Physics*. CRC Press.

**Xiong D, Douthe C, Flexas J**. **2018**. Differential coordination of stomatal conductance, mesophyll conductance, and leaf hydraulic conductance in response to changing light across species. *Plant, Cell & Environment* **41**: 436–450.

**Xiong D, Flexas J, Yu T, Peng S, Huang J**. **2017**. Leaf anatomy mediates coordination of leaf hydraulic conductance and mesophyll conductance to CO_2_ in *Oryza*. *New Phytologist* **213**: 572–583.

**Zhou H, Akçay E, Helliker BR**. **2019**. Estimation C_4_ photosynthesis parameters by fitting intensive *A*/*C*_i_ curves. *Photosynthesis Research* **141**: 181–194.

**Xiong D, Nadal M**. **2020**. Linking water relations and hydraulics with photosynthesis. *The Plant Journal* **101**: 800–815.

**Xu HM, Li YY, Shi H**. **2021**. Plasticity of leaf hydraulic conductance in maize in response to varying nitrogen and water supplies. *Russian Journal of Plant Physiology* **68**: 136–146.

**Yang S-J, Zhang Y-J, Sun M, Goldstein G, Cao K-F**. **2012**. Recovery of diurnal depression of leaf hydraulic conductance in a subtropical woody bamboo species: embolism refilling by nocturnal root pressure. *Tree Physiology* **32**: 414–422.

**Ye M, Wu M, Zhang H, Zhang Z, Zhang Z**. **2021**. High leaf vein density promotes leaf gas exchange by enhancing leaf hydraulic conductance in *Oryza sativa* L. Plants. *Frontiers in Plant Science* **12**.
